# Structural Basis for the Secretion of EvpC: A Key Type VI Secretion System Protein from *Edwardsiella tarda*

**DOI:** 10.1371/journal.pone.0012910

**Published:** 2010-09-23

**Authors:** Chacko Jobichen, Smarajit Chakraborty, Mo Li, Jun Zheng, Lissa Joseph, Yu-Keung Mok, Ka Yin Leung, J. Sivaraman

**Affiliations:** 1 Department of Biological Sciences, National University of Singapore, Singapore, Singapore; 2 Department of Biology, Faculty of Natural and Applied Sciences, Trinity Western University, Langley, British Columbia, Canada; Charité-Universitätsmedizin Berlin, Germany

## Abstract

The recently identified type VI secretion system (T6SS) is implicated in the virulence of many Gram-negative bacteria. *Edwardsiella tarda* is an important cause of hemorrhagic septicemia in fish and also gastro- and extra-intestinal infections in humans. The ***E*** *. tarda* **v**irulent **p**rotein (EVP) gene cluster encodes a conserved T6SS which contains 16 open reading frames. EvpC is one of the three major EVP secreted proteins and shares high sequence similarity with Hcp1, a key T6SS virulence factor from *Pseudomonas aeruginosa*. EvpC contributes to the virulence of *E*. *tarda* by playing an essential role in functional T6SS. Here, we report the crystal structure of EvpC from *E. tarda* PPD130/91 at a 2.8 Å resolution, along with functional studies of the protein. EvpC has a β-barrel domain with extended loops. The β-barrel consists of 11 anti-parallel β-strands with an α-helix located on one side. In solution, EvpC exists as a dimer at low concentration and as a hexamer at higher concentration. In the crystal, the symmetry related EvpC molecules form hexameric rings which stack together to form a tube similar to Hcp1. Structure based mutagenesis revealed that N-terminal negatively charged residues, Asp4, Glu15 and Glu26, and C-terminal positively charged residues, Lys161, Lys162 and Lys163, played crucial roles in the secretion of EvpC. Moreover, the localization study indicates the presence of wild type EvpC in cytoplasm, periplasm and secreted fractions, whereas the N-terminal and C-terminal mutants were found mostly in the periplasmic region and was completely absent in the secreted fraction. Results reported here provide insight into the structure, assembly and function of EvpC. Further, these findings can be extended to other EvpC homologs for understanding the mechanism of T6SS and targeting T6SS mediated virulence in Gram-negative pathogens.

## Introduction

Gram-negative bacteria use various secretion systems to transport proteins across bacterial membranes. To date, at least seven secretion systems are known in Gram-negative bacteria (type I to VII secretion systems). The type VI secretion system (T6SS) is a recently discovered protein transport complex which contributes to bacterial pathogenesis [1]–[4]. T6SS was initially identified in *Vibrio cholerae* and named IcmF associated homologous protein (IAHP) cluster [5]. In 2006, Mekalanos group showed that the IAHP gene clusters of *V. cholerae* and *Pseudomonas aeruginosa* were involved in protein secretion [6] and Pukatzki *et al*., [7] renamed this novel secretion system as T6SS.

T6SSs are present in animal and plant proteobacteria and play an important role in the virulence of many human and animal pathogens [2], [3], [4]. *In silico* analyses showed that T6SSs are found in at least 92 bacterial genomes and can be divided into four to five different phylogenetic groups [8]. Hallmarks of T6SSs include the presence of an ortholog of IcmF, an AAA^+^ ATPase and at least two secreted proteins, namely hemolysin co-regulated protein (Hcp) and valine glycine repeat protein (VgrG) [2], [3], [4], [5]. Hcp homologs are secreted through a functional T6SS, and act as virulence factors in pathogens such as *P. aeruginosa, V. cholerae,* and *Edwardsiella tarda* [7], [6], [9]. Hcp1 in *P. aeruginosa* is actively secreted in lungs of infected cystic fibrosis patients and is likely to contribute to pathogenesis [6]. The crystal structure of Hcp1 from *P. aeruginosa* revealed that it can associate into hexameric rings that stack onto each other to form a nanotube-like channel [6], [10]. Another common virulence factor secreted by T6SS is VgrG. It has a trimeric phage tail spike like structure similar to that of the T4 phage gp5(3)-gp27(3) complex (PDB 2P5Z; [11]). It is proposed that the VgrG protein might act as a membrane-puncturing device to help deliver effectors into host cells [12].

*E. tarda* is a Gram-negative pathogen which is associated with septicemia and fatal infections in a wide variety of animals including fish and humans [13], [14]. In humans, it causes gastro- and extra-intestinal infections such as myonecrosis, bacteremia, septic arthritis and wound infections [15]. Using a comparative proteomics approach, we have previously identified two secretion systems, namely type III secretion system (T3SS) and T6SS, from a fish isolate PPD130/91 [9], [16], [17], [18]. In *E. tarda*, T3SS and T6SS contributed to the replication rates inside gourami phagocytes and virulence towards gourami fish. Thus, the putative T6SS in *E. tarda* was initially named EVP (***E*** *dwardsiella tarda* **v**irulent **p**rotein). A systematic deletion of the T6SS genes in *E. tarda* revealed that 13 out of 16 genes in the EVP cluster are essential for secretion of T6SS substrates. Based on functional assays, we classified the EVP (T6SS) proteins into three groups: (1) eleven intracellular apparatus (non-secreted) proteins, (2) three secreted proteins, and (3) two proteins which are not required for the T6SS-dependent secretion. Secreted proteins of *E. tarda* T6SS include a Hcp homolog EvpC, a VgrG homolog EvpI, and a novel protein EvpP which is not commonly present in other T6SS clusters [9]. Our previous functional studies showed that the major secreted protein EvpC is a key member in the EVP system of *E. tarda* and the secretion of EvpC is a critical step for the function of T6SS [9], [17]. In this work, we focus on eliciting the secretion property of EvpC from structural and functional aspects.

Here we report the crystal structure of EvpC at 2.8 Å resolution and studies on its oligomerization and secretion. Gel filtration and analytical ultra centrifugation (AUC) analyses confirmed that the oligomerization state of EvpC is concentration dependent. Further, structure-guided mutagenesis, followed by functional studies, demonstrated that N-terminal negatively charged residues, Asp4, Glu15 and Glu26, and C-terminal positively charged residues, K161, K162 and K163, are involved in secretion of EvpC. The localization study on EvpC and its mutants in the cytoplasm, periplasm and secreted fraction showed the presence of wild type EvpC in all three regions, whereas the N-terminal triple mutant and C-terminal mutant was predominantly found in the periplasmic region and none in the secreted fraction. The structural basis for the secretion of EvpC reported here can be extended to its homologs in other pathogens and, subsequently, this will help to understand the T6SS mechanism in Gram-negative bacteria.

## Results

### Overall structure

**Figure 1:**
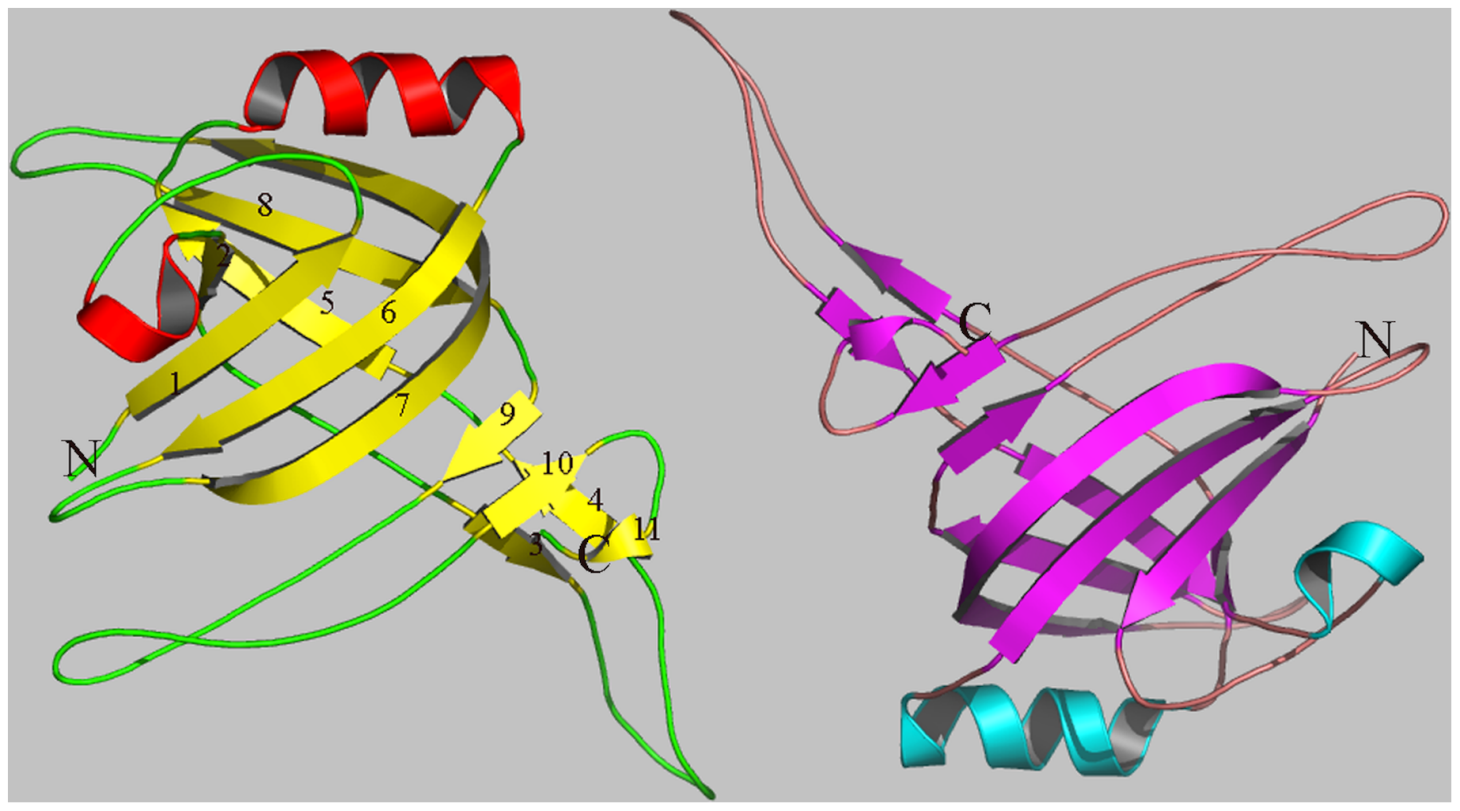
Ribbon diagram of the two EvpC molecules of an asymmetric unit. The N- and C- termini are indicated. This figure and the following figures in the paper were prepared using PyMol [40].

The structure of recombinant EvpC from *E. tarda* was solved by molecular replacement using a dataset collected on an R-axis rotating anode generator and refined to a 2.8 Å resolution (Table 1). The EvpC model consists of residues from Ala2 to Lys163. There are two molecules in the asymmetric unit (Figure 1). These two molecules are part of two hexameric rings which can be generated using crystallographic symmetry. Each monomer of EvpC consists mainly of a β-barrel domain, with extended loops lying predominantly on one side of the β-barrel. The diameter of the monomeric β-barrel is approximately 12 Å consisting of 11 anti-parallel β-strands. In addition, an α-helix (Ala66 - Gln76) is located on one side of the β-barrel. Residues Gly35 to Met53 form an extended loop which protrudes approximately 20 Å from the β-barrel (Figure 1).

**Table 1:** Data Collection and Refinement Statistics.

**Data collection**	
Space group	P6
Cell dimensions	
*a*, *b*, *c* (Å)	85.5, 85.5, 93.1
α, β, γ (°)	90, 90, 120
Resolution range (Å)	50.0 - 2.8 (2.9 - 2.8)
a *R*_sym_	16.3(33.7)
*I*/σ*I*	7.5
Completeness (%)	94.2(88.5)
Redundancy	7.1(3.4)
**Refinement**	
Resolution (Å)	29 - 2.8
No. reflections	9074
b *R*_work_/c *R*_free_	0.238/0.284
**Number of atoms**	
Protein	2348
Water	71
***B*** **-factors**	
Protein	26.4
Water	26.8
**R.m.s deviations**	
Bond lengths (Å)	0.009
Bond angles (°)	1.250

a R_sym_  =  Σ|Ii-<I>| /Σ|Ii| where Ii is the intensity of the i^th^ measurement, and <I> is the mean intensity for that reflection.

b R_work_  =  Σ|F_obs_ - F_calc_|/Σ|Fobs| where F_calc_ and F_obs_ are the calculated and observed structure factor amplitudes, respectively.

c R_free_  =  as for Rwork, but for 5.0% of the total reflections chosen at random and omitted from refinement.

Values in parentheses correspond to the highest resolution shell.

### Homology with other Hcp1 family proteins

**Figure 2:**
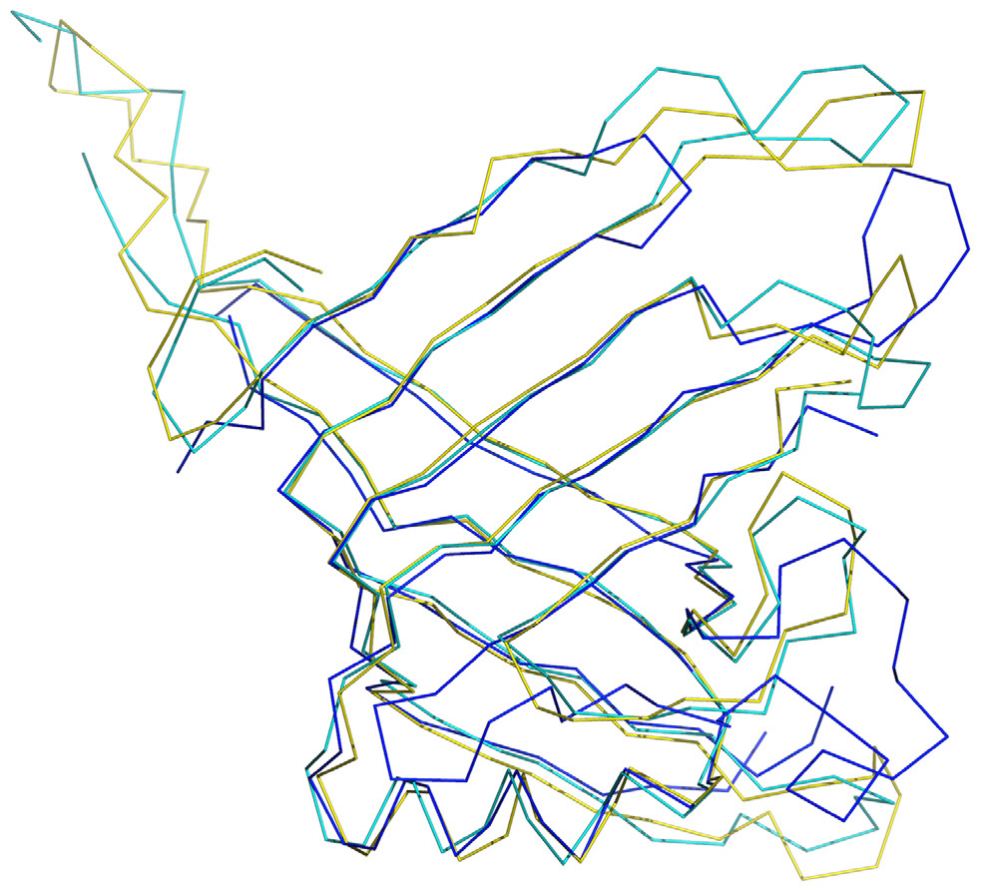
Cα superposition of EvpC with homologue structures Hcp1 and Hcp3. EvpC is in yellow, Hcp1 in cyan and Hcp3 is in blue color.

**Figure 3:**
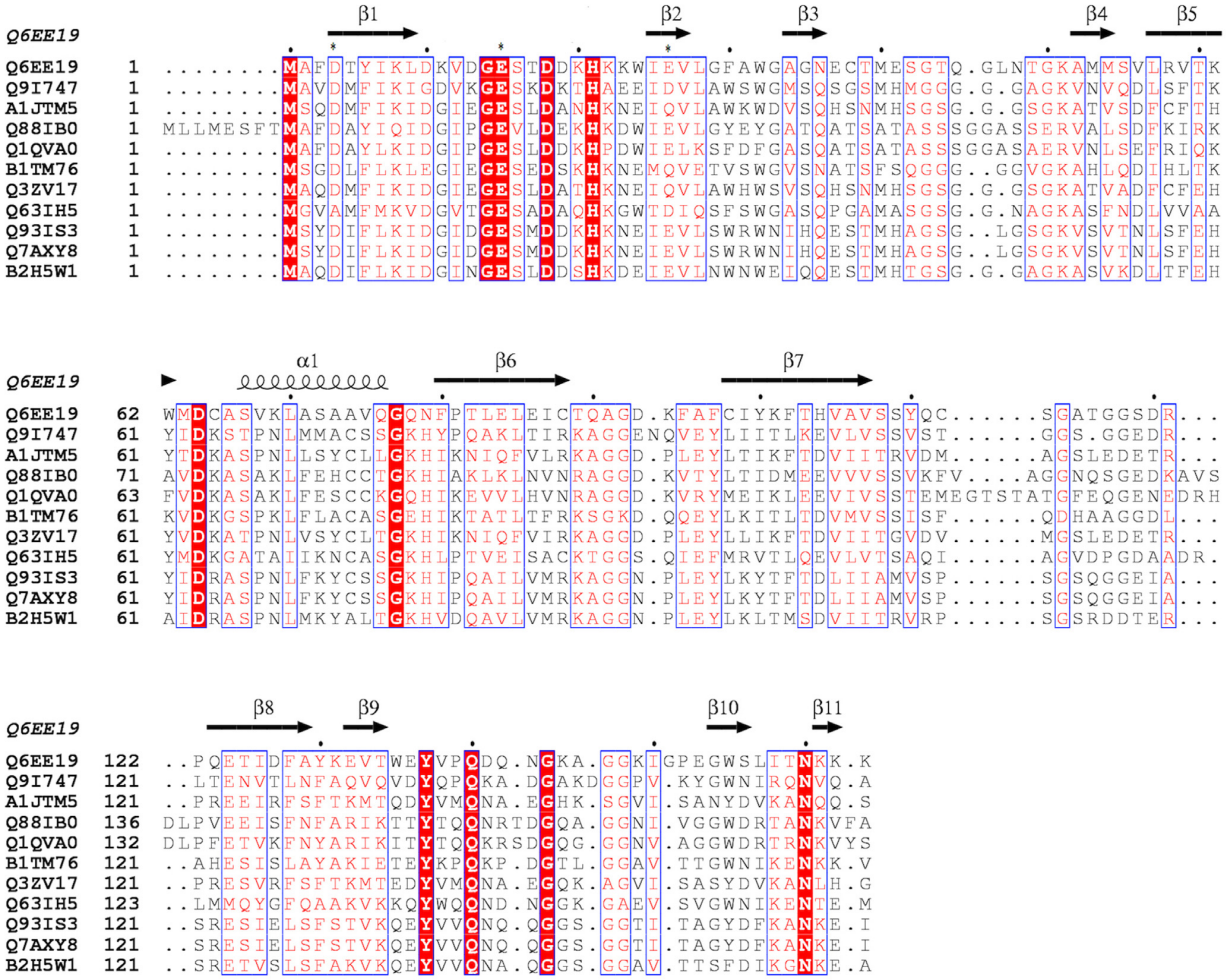
Structural and sequence alignment of EvpC. First and second rows: Structure based sequence alignment of EvpC (PDB code 3EAA; Q6EE19), Hcp1 (PDB code 1y12, Q9I747). Strictly conserved residues are shaded red with semi-conserved residues lettered in red. Three to eleven rows: Suffix: Q6EE19: EvpC, *E. tarda;* Q9I747: Hcp1, *Psuedomonas aeruginosa*; A1JTM5: Hypothetical protein, *Yersinia enterocolitica*; Q88IB0: Hypothetical protein, *Pseudomonas putida*; Q1QVA0: Hypothetical protein, *Chromohalobacter salexigens*; B1TM76: Type VI secretion system effector, *Methylocella silvestris*; Q3ZV17: Hypothetical protein, *Enterobacter sakazakii*; Q63IH5: Hypothetical protein, *Burkholderia pseudomallei*; Q93IS3: Putative cytoplasmic protein, *Salmonella typhimurium*; Q7AXY8: SciM protein, *Salmonella enterica*; B2H5W1: SciM protein, *Burkholderia pseudomallei* 1655. Residues of EvpC mutated in these studies are denoted by asterisks. Sequence alignment was done by ClustalW [41] and the figure was prepared using ESPript [42].

A search for topologically similar proteins within the PDB was performed with the program DALI [19]. Significant structural similarities were observed between EvpC and the proteins Hcp1 (PDB code 1y12; rmsd = 1.6 Å for 158 Cα atoms; 32% sequence identity) and Hcp3 (coordinates recently deposited; not yet described in the literature; PDB code 3HE1; rmsd = 2.2 Å for 130 Cα atoms; 17.7% sequence identity) from *P. aeruginosa*, most notably the presence of an overall β-barrel architecture. Figure 2 shows the Cα superposition of EvpC, Hcp1 and Hcp3. Significant differences are observed between Hcp3 and EvpC. Particularly, several loops of Hcp3 are different in length and conformation, compared to both Hcp1 and EvpC. The structure based sequence alignment of EvpC with Hcp1 from *P. aeruginosa,* and sequence alignment with other Hcp homologs from various representative pathogenic bacterial species/strains is given in Figure 3. The overall structural and sequence similarity between EvpC and Hcp1 strongly suggest that EvpC is a member of the Hcp family of proteins. Recently, Pell *et. al,* [20] (PDB 2K4Q) reported that the Hcp-like proteins are structurally related to the major tail proteins of enterobacteriophage lambda. The sequence identity of EvpC with various homologous proteins from selected pathogenic bacterial species is given in Table S1.

A BLAST search [21] with the sequence of EvpC revealed that it has more than 100 homologs from various bacterial species or strains. All of these homologs shares approximately 50% similarity and ∼35% sequence identity with EvpC (Figure S1). Sequence conservation is spread throughout the molecule, particularly at the N- and C-termini, as well as MDCAS and VAVS sequence motifs in the middle of the protein. However, no functional studies are available to reveal the importance of these conserved motifs.

### Oligomerization

**Figure 4:**
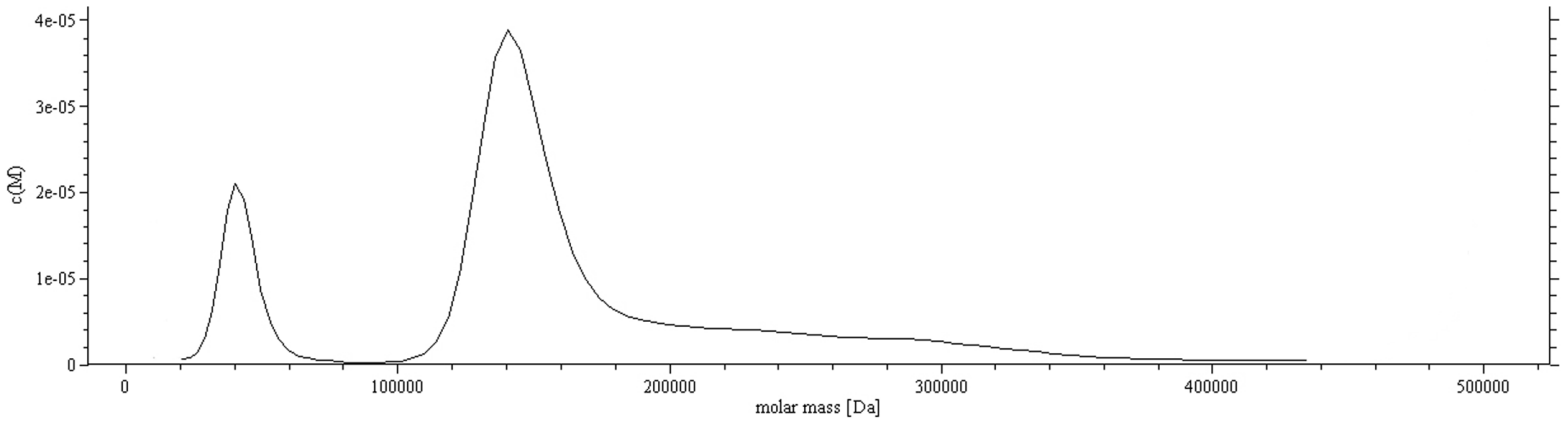
Analytical ultra centrifugation profile of EvpC on the sample eluted (∼2 mg/ml concentration) from the gel filtration column (Hiload 16/60 Superdex 200). This shows a mixture of two oligomers. A small peak with an apparent molecular weight of 40 kDa (dimer) and a larger peak around 120 kDa (hexamer).

**Figure 5:**
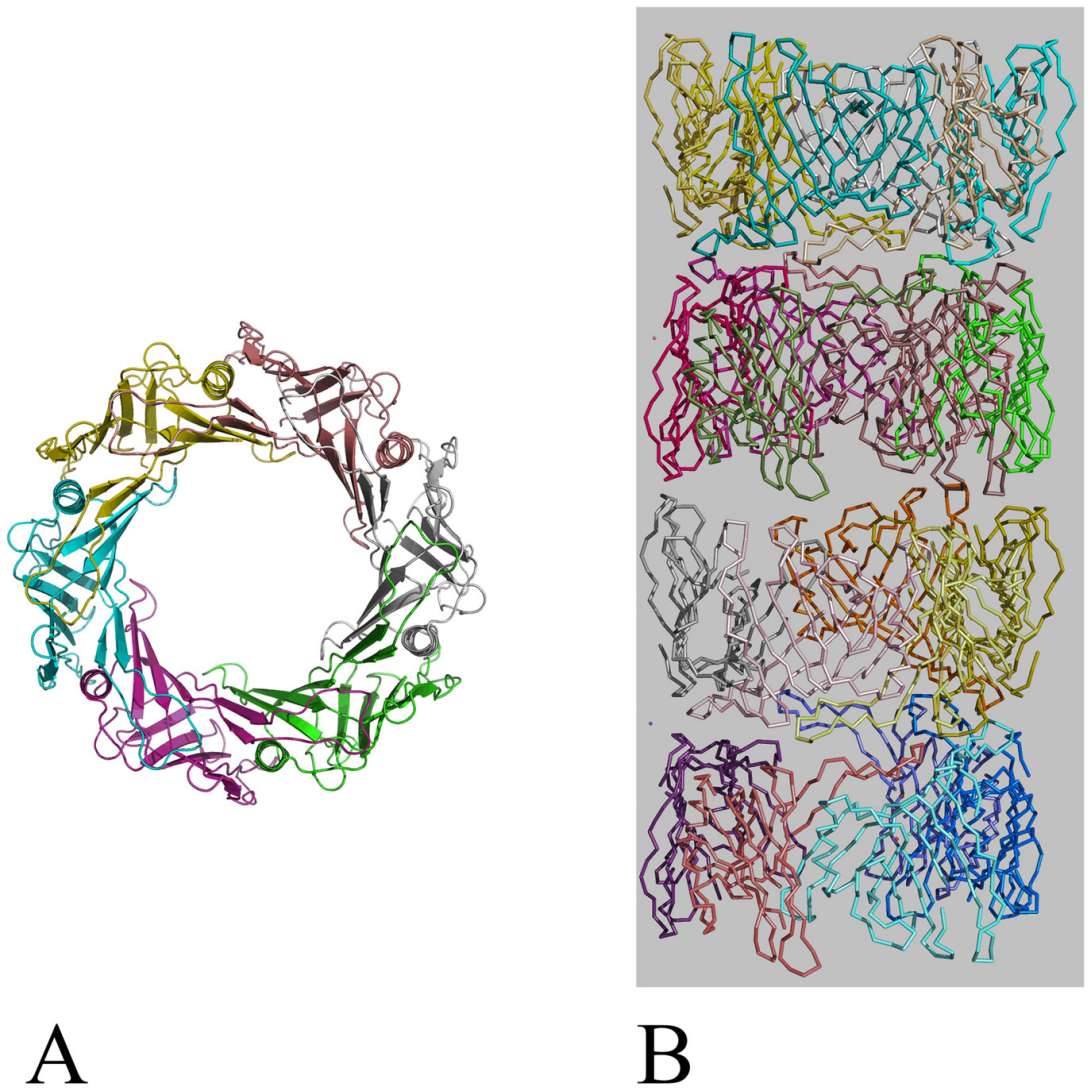
Hexameric EvpC generated by symmetry related molecules. (A) Ribbon representation of EvpC hexameric ring formed by the symmetry related molecules. Diameter of the pore is approximately 40 Å. (B) Stacking of EvpC hexameric rings which form a tube-like architecture.

The oligomerization state of EvpC in solution was investigated using AUC, dynamic light scattering (DLS) and gel filtration. AUC analysis of the eluted protein (∼ 2 mg/ml) from the Ni-NTA beads showed two peaks corresponding to an apparent molecular weight of a dimeric (40 kDa) and hexameric (120 kDa) EvpC. The hexamer is the predominant form of EvpC, which is further evident from the c(M) values (Figure 4). Gel filtration of the concentrated protein (>2 mg/ml) produced a single peak corresponding to hexameric EvpC (Figure S2). At a low concentration of EvpC (∼0.8 mg/ml) gel filtration revealed two species corresponding to the hexamer and a predominant dimeric form (Figure S3). This observation is supported by AUC (data not shown). DLS of concentrated EvpC (6 mg/ml) showed an apparent molecular weight of 212 kDa corresponding to two hexamers of EvpC. It should be noted that the molecular weight estimated from DLS experiments may not be accurate. DLS usually gives the particle size of the predominant species. Precise determination of the molecular weight with DLS is extremely difficult given that EvpC forms hexameric rings rather than globular particles. Although concentration dependent oligomerization (dimer and hexamer) of EvpC was observed in solution, all of our experiments showed that hexamer is the preferred oligomeric form of EvpC. A head-to-head stacked hexameric ring structure of EvpC can be observed in the crystal lattice (Figure 5A). This stacked ring structure forms a tube with an outer diameter of 80 Å and an inner diameter of 40 Å (Figure 5B). In Hcp1, the asymmetric unit consisted of 3 monomers and the hexameric ring could be generated by 6-fold symmetry. These symmetry related hexameric rings stacked in top-to-bottom fashion forming an extended nanotube [10]. In EvpC the asymmetric unit consists of two monomers and the 6-fold symmetry generated two hexameric rings stacked in head-to-head fashion. We assume that this head-to-head stacking might be a crystallographic artifact. Taken together, the observed hexameric form of EvpC in solution and the hexameric ring formation by the symmetry related EvpC molecules in the crystal, we speculate that the tube formation by EvpC is analogous to Hcp1 and functionally relevant.

### N terminal negatively charged residues are critical for EvpC secretion

**Figure 6:**
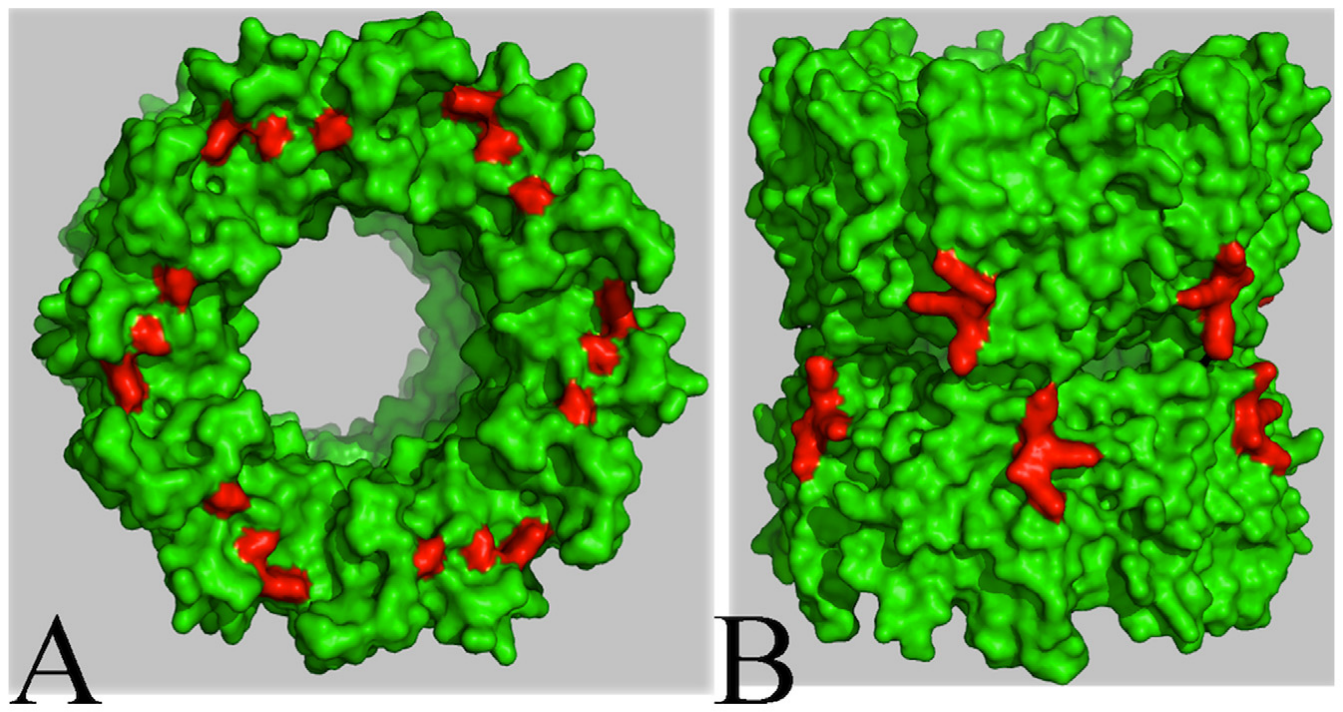
Surface representation of EvpC hexameric ring. (A) Location of N-terminal negatively charged residues (red) (top view). (B) Location of the surface exposed C-terminal residues (red) (side view). The residues targeted for the mutational studies are shown in red.

**Figure 7:**
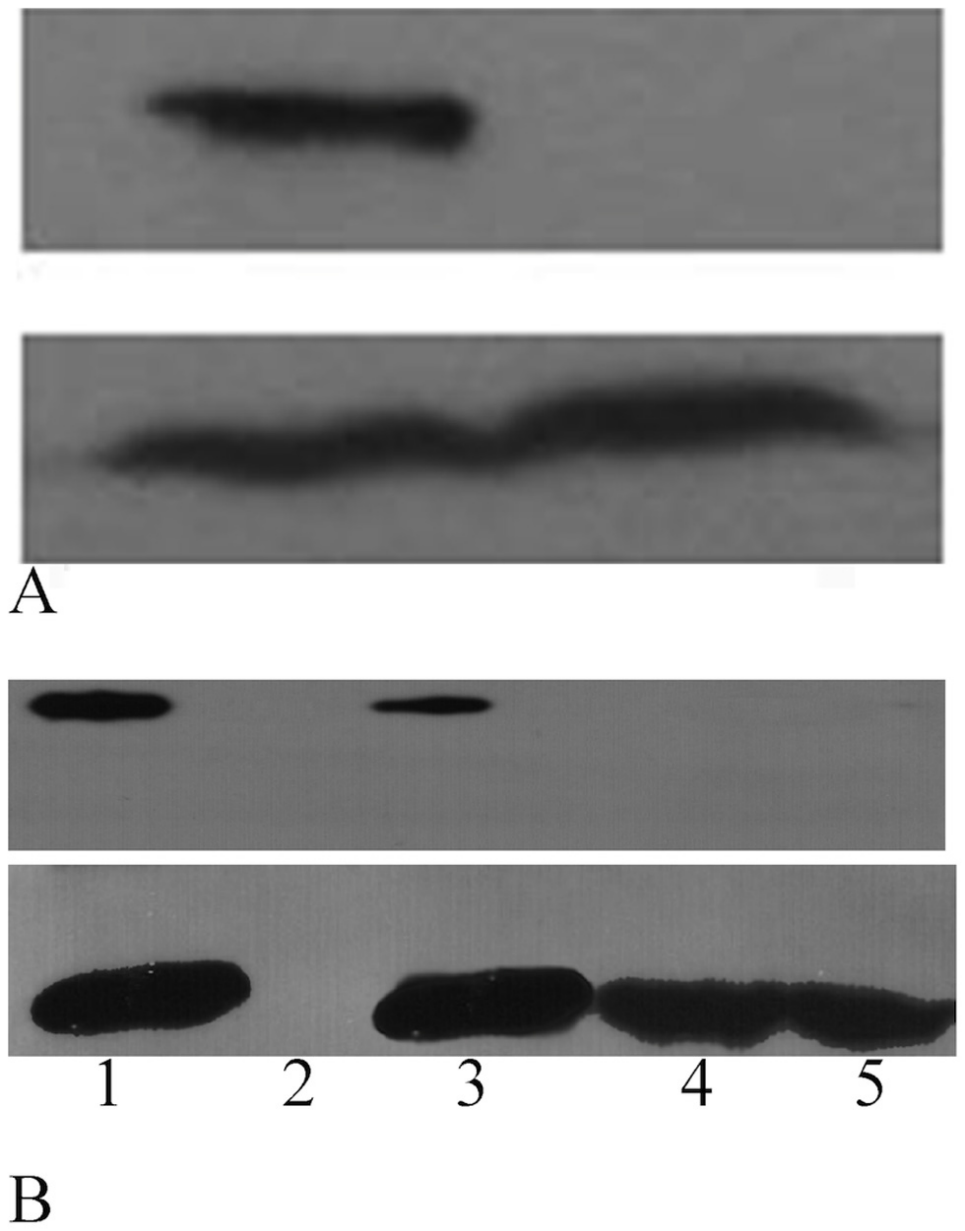
Western blots of the expression and secretion of EvpC from *E. tarda*. EvpC mutants harboring pSA10-EvpC wild type and various mutants used in our study. (A) ΔevpC expressing pSA-evpC and pSA-evpC-6His. Expression (bottom panel) and secretion (top panel). The secretion of EvpC is completely abolished by adding 6His-tag at the N-terminus despite having similar expression level as wild type. (B) Expression (bottom panel) and secretion (top panel) of EvpC in wild type, N-terminal and C-terminal mutants of EvpC. 1. Wild type 2. ΔevpC 3. Δevpc + evpC 4. Δevpc + evpC (D4AE15AE26A) 5. Δevpc + evpC (M1-N160).

EvpC homologous proteins from T6SS (such as Hcp) do not contain classical signal peptides, indicating that they are not secreted in a way similar to Sec or type II secretion systems [22], [23]. It has been suggested that T6SS may use a unique but unknown mechanism to export its proteins, which probably cross the bacterial cell membrane in a single step [24]. From the crystal structure of EvpC, we have identified 6 negatively charged residues in the first 26 amino acids that are clustered in the N-terminal region (Figure 6A). Notably, a BLAST search showed the presence of 3 to 7 negatively charged residues at the N-terminal region of EvpC homologs. Three of these residues are conserved among 50 homologs, suggesting the importance of this cluster (Figure S1). In addition, we found that introducing a 6His tag at the N-terminal of EvpC completely blocked its secretion (Figure 7A). To investigate the importance of these negatively charged residues in the N-terminal region of EvpC, a series of EvpC single, double and triple site-directed mutants was created by substituting D4, E15 and E26 with Ala (Table 2). Plasmids carrying genes of these EvpC mutants were used to transform an *E. tarda* Δ*evpC* mutant and their respective expression and secretion of EvpC were anlaysed. The secretion of EvpC in various EvpC mutants was similar to wild type (Figure S4), except for the triple mutant D4A E15A E26A (Figure 7B), which seriously impaired EvpC secretion in spite of having comparable growth rate with that of the wild type bacteria carrying the intact evpC gene (Figure 7B). The total cell protein profile (Figure 7B) confirmed the expression of EvpC in this triple mutant suggesting that the negatively charged residues D4, E15 and E26 together play a key role only in the secretion of EvpC but not in its expression.

**Table 2:** The bacteria strains and plasmids used in this study.

Strain or plasmid	Description	Reference or source
Strains		
PPD130/91	Wild-type Edwardsiella tarda, Kms, Colr. Amps	Ling *et al*. (2000)
ΔevpC	PPD130/91, in-frame deletion of evpC	Srinivasa *et al*. (2004)
Plasmids		
pSA10	pKK177-3 derivative, *lacI*, Amp^r^	S. Altuvia
pSA*evpC*	pSA10 with *evpC* fragment	This study
pSA6His-*evpC*	pSA10 with *evpC* fragment and 6His tag at N terminus	This study
pSA*evpC*D4A	pSA10 with *evpC* fragment, D4 residue is substituted with Ala	This study
pSA*evpC* E15A	pSA10 with *evpC* fragment, E15 residue is substituted with Ala	This study
pSA*evpC* E26A	pSA10 with *evpC* fragment, E26 residue is substituted with Ala	This study
pSA*evpC* D4AE15A	pSA10 with *evpC* fragment, D4 and E15 residues are substituted with Ala	This study
pSA*evpC* D4AE26A	pSA10 with *evpC* fragment, D4 and E26 residues are substituted with Ala	This study
pSA*evpC* E15AE26A	pSA10 with *evpC* fragment, E15 and E26 residues are substituted with Ala	This study
pSA*evpC* D4AE15AE26A	pSA10 with *evpC* fragment, substitute residues D4, E15 and E26 residues are substituted with Ala	This study
pSA*evpC* 1–160	pSA10 with *evpC* fragment, with K161K162K163 deletion	This study
pETM	pET32 derivative, Ampr	Novagen
pETM*evpC*	pETM with *evpC* fragment and 6His tag at C terminus,	This study
pETM*evpC* D4AE15A	pETM with *evpC* fragment and 6His tag at C terminus, D4 and E15 residues are substituted with Ala	This study
pETM*evpC* D4AE26A	pETM with *evpC* fragment and 6His tag at C terminus, D4 and E26 residues are substituted with Ala	This study
pETM*evpC* E15AE26A	pETM with *evpC* fragment and 6His tag at C terminus, E15 and E26 residues are substituted with Ala	This study

### Role of C-terminal positively charged residues in EvpC secretion

**Figure 8:**
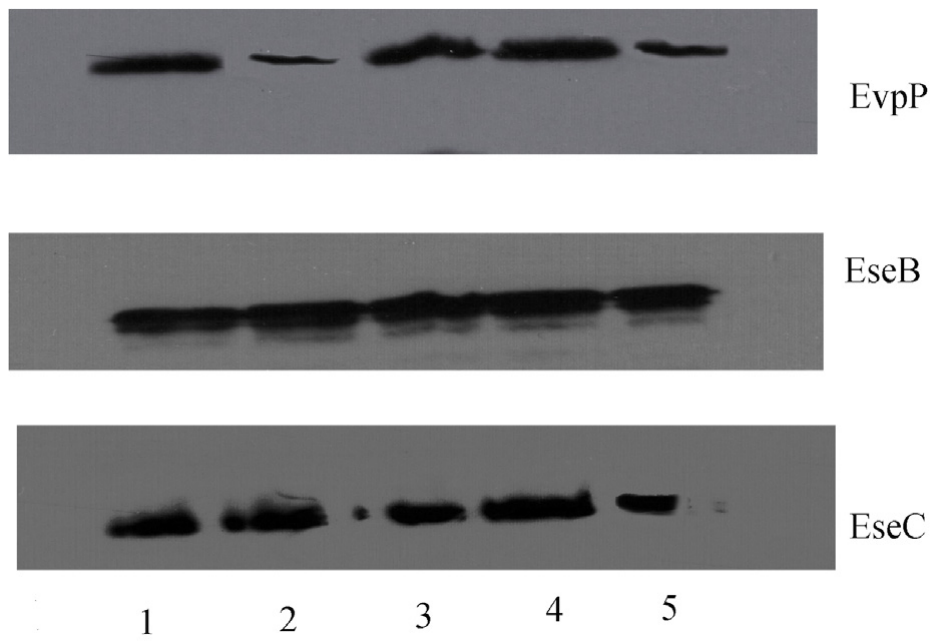
Secretion of EvpP, Ese-B and EseC in wild type, N-terminal mutant and C-terminal mutant. **1.** Wild type 2. ΔevpC 3. Δevpc + evpC 4. Δevpc + evpC (D4AE15AE26A) 5. Δevpc + evpC (M1-N160). The western blot was probed with anti-EvpP, anti-EvpC, anti-EseB and anti-EseC rabbit polyclonal antibodies.

Positively charged amino acids have been shown to be important in targeting proteins into secretory pathways and facilitating protein secretion [25]–[27]. EvpC has a cluster of positively charged residues located at the C-terminal region (Figure 6B). In order to investigate the possible involvement of these basic residues (K161, K162 and K163) in the secretion of EvpC, we created the EvpC C-terminal deletion (EvpC M1-N160) mutant. Subsequently, secretion assays were performed to probe the impact of these residues on the secretion of T6SS proteins, in particular EvpC. Western blot analysis showed that the secretion of EvpC was seriously impaired with this C-terminal deletion mutant (Figure 7B). The total cell protein profile (Figure 7B) was comparable in the wild type as well as C-terminal and N-terminal mutants which indicates that the expression of EvpC was unaffected by these mutations. In addition we have observed that there was a significant reduction in the secretion of EvpP due to the C-terminal mutation (Figure 8). These results strongly suggest that the positively charged cluster of amino acids in the C-terminal region of EvpC is important for the secretion of EvpC and EvpP.

Our results also showed that none of these EvpC mutants exhibited any defect in the secretion of EseB and EseC, two known secreted translocators in the T3SS (Figure 8). To further verify the properties of EvpC mutants, we have expressed double, triple and C-terminal deletion mutants of EvpC and tested their oligomerization. Gel filtration experiments showed that all of these mutants exhibit the same oligomeric form as wild type EvpC (Figure S5).

### Localization of EvpC

**Figure 9:**
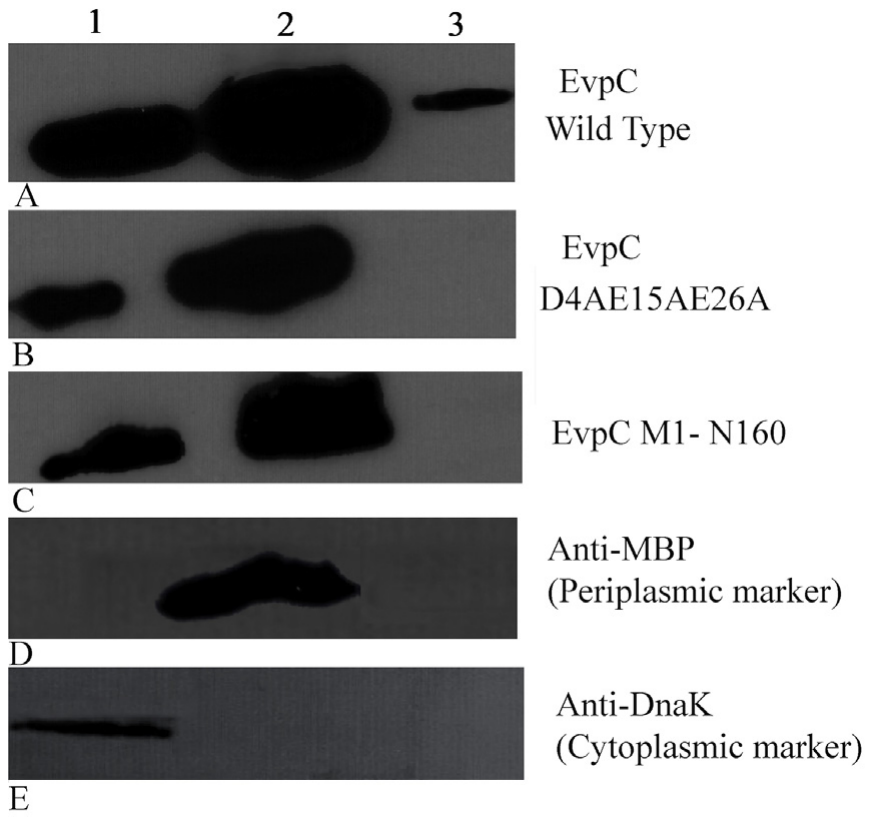
Western blot analysis of the localization of EvpC using anti-EvpC antibody. (A) Wild type (B) N-terminal triple mutant (C) C-terminal mutant (D) Periplasmic marker (E) Cytoplasmic marker. EvpC is predominantly localized in the periplasmic space and is secreted outside only by the wild type bacteria. 1. Cytoplasmic fraction 2. Periplasmic fraction 3. Secreted fraction. Anti-MBP Monoclonal antibody (NEB) and Anti-DnaK Monoclonal antibody (Stressgen) were used as periplasmic and cytoplasmic markers.

In order to verify the localization of EvpC we carried out mechanical fractionation studies. Western blot analysis confirmed the presence of wild type EvpC in the cytoplasm, periplasm and in the secreted fractions, whereas in the N-terminal triple mutant and C-terminal deletion mutant, EvpC was completely absent in the secreted fraction (Figure 9). Notably in both of these mutants, EvpC was predominantly localized in the periplasm which confirms the expression of protein. Moreover the use of monoclonal antibodies against DnaK (cytoplasmic marker) and MBP (periplasmic marker) confirm that there was no cross-contamination between cytoplasmic, periplasmic and secreted fractions (Figure 9). The absence of EvpC in the secreted fraction in spite of its normal expression clearly reveals a serious defect in the secretion of these mutants.

## Discussion

Hcp homologs are the major secreted proteins of T6SS in many Gram-negative bacteria including *P. aeruginosa*, *S. enterica, V. cholera*, and *E. tarda* [2]. Mutations of Hcp homologs decrease the virulence of pathogenic bacteria [7], [6], [17]. Thus, the role of Hcp homologs is vital for the function of T6SS. However, the secretion and transport mechanisms of Hcp homologs remain unknown. EvpC is essential for a functional T6SS and plays an important role in the virulence of *E. tarda* [9], [17]. Deletion of EvpC leads to lower replication rates in phagocytes and increased LD_50_ in blue gourami fish by two log value [17]. The structure of EvpC reported here is similar to that of Hcp1 from *P. aeruginosa.* Mougous *et al* [6] revealed the presence of Hcp1 in the lungs of *P. aeruginosa* infected patients. Moreover, it has been shown using the *in vitro* experiments that Hcp1 can be assembled to form nanotube like structures, which contain upto 25 subunits and are around 100 nm in length [10]. In the case of EvpC, symmetry related molecules forms hexameric rings and these rings stacks to form nanotube-like structures.

Our studies on the oligomeric state of EvpC using gel filtration and AUC demonstrate the presence of two oligomeric states. We found that EvpC exists as a dimer at low concentrations and as a hexamer at higher concentrations. Based on these findings we speculate that the concentration-dependent oligomerization of EvpC may have functional implications. EvpC may exist as dimers after production, and subsequently is transported to the assembly point during infection where a higher concentration of EvpC might facilitate the formation of hexameric rings to constitute the secretion apparatus.

Structure guided mutational studies showed that the N-terminal negatively charged and C-terminal positively charged residues can affect the secretion of EvpC. In addition, the presence of 6His tag at the N-terminal of EvpC adversely affected its secretion, possibly due to the interactions between the His-tag and these negatively charged residues. The analysis of crystal structure confirms that all these residues (D4, E15, E26, K161, K162 and K163) are exposed on the outer rim of the EvpC hexameric ring which makes them available for interaction with other proteins.

The secretion of EvpC was seriously impaired in both N-terminal (D4AE15AE26A) and C-terminal mutants (Figure 7B). The secretion of EvpP was considerably reduced in the C-terminal mutant (EvpC M1-N160). On the other hand, no reduction in the secretion of EvpP was observed in the N-terminal triple mutant (Figure 8). This indicates that the N-terminal residues are only important for the secretion of EvpC whereas the C-terminal residues play a role in the secretion of EvpC as well as for other T6SS proteins like EvpP. The total cell protein analysis showed that the expression of C-terminal mutant and the N-terminal triple mutant of EvpC was comparable to the wild type bacteria suggesting that these mutants only cause defects in the secretion of EvpC but not its expression (Figure 7B). Further the fractionation studies confirmed this finding (Figure 9). We noticed an increased amount of EvpC in the periplasm compared to the cytoplasm in these mutants which can be attributed to the failure in secretion. The mechanical fractionation studies suggest that the N-terminal and C-terminal residues are involved in the transport of T6SS proteins from the periplasm to the extracellular region. It has been shown that the C-terminal region of Type IV and Type V secretion system proteins are involved in protein secretion [26]–[28]. Mougous *et al* [24] showed that the EvpC homolog, Hcp1 is temporarily localized in periplasm, where it assembles into nanotubes (stacked hexamers) and may be associated with VgrG (a EvpI homolog) at the tip to puncture the membrane and allow tube extension outwards into the extracellular space [4], [24]. We previously reported that secretion of EvpC and EvpI are mutually dependent and mutation of either one of them leads to the absence of EvpP in the supernatant [9]. Based on our mechanical fractionation study and secretion assay it is tempting to speculate that the interactions between EvpC and EvpI might be disrupted in these mutants causing the abolition of secretion. Notably, both the N-terminal and C-terminal residues are exposed in the hexameric tube of EvpC which makes them available for engaging with other molecules (Figure 6A and 6B). Moreover our results showed that none of these EvpC mutants showed any defect in the secretion of T3SS proteins such as EseB and EseC (Figure 8). Thus EvpC being one of the secreted proteins of T6SS does not seem to cross-talk with secreted proteins involved in T3SS.

In conclusion, EvpC homologs are widely found among a number of pathogenic bacterial species and represent a new class of secreted apparatus proteins. Understanding the role of EvpC will provide a significant step forward in resolving the T6SS mechanism. This study uncovers the critical role of negatively charged N-terminal residues (Asp4 Glu15 and Glu26) and the C-terminal positively charged residues in the secretion of EvpC. Impairing the secretion of EvpC will decrease the virulence of the pathogenic bacteria. This may lead to the development of novel strategies to restrict the T6SS-containing pathogenic bacteria.

## Materials and Methods

### Plasmid and strain construction

The *evpC* gene was PCR-amplified from *E. tarda* genomic DNA and cloned into a derivative of pET vector (pETM32) (Novagen) or pSA10 vector. *evpC* mutant plasmids were derived from wild type *evpC* from pSA-*evpC* and pET-*evpC* by site-directed mutagenesis using inverse PCR or overlap PCR techniques. Various pSA-*evpC* mutant plasmids were used to transform *E. tarda* Δ*evpC* strain to yield different EvpC expression strains. The bacterial strains and plasmids used in this study are listed in Table 2.

### Purification and crystallization

Plasmid DNA was used to transform *E. coli* BL21 and cells grown in defined M9 medium [29] supplemented with 25 mg/L-SelMet, at 37°C to 0.6 AU at OD_600._ One liter of culture was induced with 100 µM IPTG and continued to grow at 20°C overnight. Cells were then harvested by centrifugation (9000 g; 20 min, 4°C) and resuspended in 40 ml of lysis buffer (50 mM Tris-HCl, pH 7.5, 0.2 M NaCl, 1 mM EDTA, 1% (w/v) Triton X-100, 5% (w/v) glycerol, 2 mM DTT and one protease inhibitor tablet (Roche Diagnostics)). The protein was purified in three steps, using DEAE-Sepharose (Amersham Pharmacia), Ni-NTA (Qiagen) and Gel Filtration (Superdex200) columns. The purified protein was kept in a buffer consisting of 20 mM Tris-HCl pH 7.5, 200 mM NaCl, 2 mM DTT, and 5%( w/v) glycerol.

Crystallization conditions for the protein were screened using Index Screens (Hampton Research) using the hanging-drop vapor-diffusion technique at 21°C. Crystallization drops containing 1 µl protein solution (40 mg/ml) and 1 µl reservoir solution. Diffraction quality crystals grew from a reservoir solution consisting of 0.2 M Ammonium sulfate, 0.1 M Bis-Tris pH 6.5 and 25% Polyethylene glycol 3350. Crystals belonged to the space group P6 with two molecules in the asymmetric unit. The unit cell parameters were a =  85.5, b = 85.5, c = 93.1 Å (Table 1) and the solvent content was 40% (Matthews constant 2.16 Å^3^/Da)[30].

### Data collection, structure solution and refinement

Crystals were cryoprotected in the reservoir solution supplemented with 12.5% Polyethylene glycol 3350 and flash cooled at 100K. A complete diffraction dataset were collected using Rigaku X-ray generator mounted with an RAXIS IV^+^ image plate detector. The collected dataset was processed with HKL2000 [31]. Structure was solved by molecular replacement technique using PHASER program from the CCP4suite [32], [33]. Hcp1 protein (pdb code 1Y12, sequence identity = 32%) was used as the search model. Model building was performed using the O program [34] and refinement was carried out using Phenix [35]. Finally 123 well-defined water molecules were added, and refinement was continued until the R-value converged to 0.238 (R_free_ = 0.284) for reflections I>σ (I) to 2.8 Å resolution. The model had good stereochemistry, with all residues within the allowed regions of Ramachandran plot (Table 1) analysed by PROCHECK [36]. The 2*Fo-Fc* electron density map is shown in Figure S6.

### Preparation of Cytoplasmic, Periplasmic and Secreted protein fractions

Overnight cultures of *E. tarda* in DMEM were diluted 1∶200 in fresh DMEM and incubated for 24 hr at the indicated temperatures. For isolation of ECPs (Extra-cellular proteins), bacterial cells were stored from the culture by centrifugation (5,500×*g*, 20 min, 4°C), and the supernatant was filtered through a 0.22 µm pore size small-protein binding filter (Millipore). The ECP fraction was isolated by trichloroacetic acid (TCA) precipitation and the protein pellet was washed thrice with −20°C acetone and then air dried. Periplasmic proteins were prepared by osmotic shock treatment as described in [37] with modification. The stored cell pellets were resuspended in a 5 ml buffer containing 20% sucrose with 20 mM of Tris-HCl pH 7.5 and Na,EDTA (1_ST_ BASE). The osmotically fragile cells were collected after a static incubation for 30 min at room temperature by centrifugation at 13,000 g for 30 min. The cell pellet was then resuspended in 2 ml distilled H_2_O (4°C) and incubated with gentle agitation at 4°C for 30 min. The supernatant fraction referred to as the periplasmic fraction was collected after a centrifuge of 13,000 g at 4°C for 30 min. After the extraction of periplasmic protein, the residual cell pellets were sonicated to obtain cytoplasmic proteins. Both the cytoplasmic and periplasmic fractions were precipitated with TCA and the protein pellet was washed thrice with −20°C acetone and then air dried. The protein pellets of ECPs were solubilized in 20 µl of Ready Prep reagent 3 (5 M urea, 2 M thiourea, 2% (w/v) CHAPS, 2% (w/v) SB 3–10, 40 mM Tris, and 0.2% (w/v) Bio-Lyte 3/10 ampholyte (Bio-Rad)) and was stored at −80°C until analysis. For the cytoplasmic and periplasmic fractions, 100 µl of Ready Prep reagent 3 were used to solubilize the proteins. The protein concentration was determined by a Bio-Rad protein assay kit with bovine serum albumin as the standard.

### Western blot analysis

Proteins were separated on 12% SDS-polyacrylamide gels. For Western analysis, proteins were transferred to PVDF membrane with a semi-dry system and examined by using the SuperSignal WestPico Chemiluminescent substrate under conditions recommended by the manufacturer (Pierce). EseB, EseC, EvpP and EvpC were detected by the addition of diluted anti-EseB (1∶10000), anti-EseC (1∶10000), anti-EvpP (1∶5000) and anti-EvpC (1∶5000) polyclonal antisera, respectively, followed by a 1∶5000 dilution of mouse anti-rabbit IgG HRP (Santa Cruz Biotechnology). Anti-MBP Monoclonal antibody (NEB) and Anti-DnaK Monoclonal antibody (Stressgen) were used as periplasmic and cytoplasmic markers.

### Dynamic light scattering (DLS)

DLS measurements were performed at room temperature on a DynaPro (Protein Solutions) DLS instrument. The percentage of polydispersity was 5% and the SOS error was 2.11 for the protein sample at 6 mg/ml concentration. The quality of the data is represented in the sum of squares (SOS) error statistic reported for each sample acquisition (a single correlation curve).

### Analytical Ultra Centrifugation (AUC)

The oligomeric state of EvpC was investigated by monitoring its sedimentation properties in sedimentation velocity experiments. 500 µl of samples at 2.5 mg/ml in TRIS buffer (Tris-HCl 10 mM pH 7.0, 200 mM NaCl) was used for these experiments. The experiments were carried out in duplicates with the presence and absence of DTT (21mM) in the buffer to find out the effect of DTT in the oligomerization state. The sedimentation velocity profiles were collected by monitoring the absorbance at 280 nm. The samples were centrifuged at 40,000 rpm at 20°C in a Beckman Optima XL-I centrifuge fitted with a six-hole AN-60 rotor and double-sector aluminum centerpieces and equipped with absorbance optics. The scans were analysed using Sedfit program [38].

### Accession Number

Coordinates of EvpC have been deposited in the Protein Data Bank (http://www.pdb.org) [39] under accession code 3EAA.

## Supporting Information

Table S1 The sequence identity of EvpC with various homologous proteins from selected pathogenic bacterial species.
(0.03 MB DOC)

Figure S1 Sequence alignment of EvpC and its homologues. The alignment was performed using ClustalW [41], and this figure was prepared using ESPript [42].
(0.02 MB PDF)

Figure S2 Gel filtration (Hiload16/60 Superdex 75 column) profile of EvpC at a higher concentration (>2 mg/ml), the peak corresponds to an apparent molecular weight of 120 KDa (hexamer).
(1.24 MB TIF)

Figure S3 Gel filtration (Hiload16/60 Superdex 75 column) profile of EvpC at a low concentration (∼0.8 mg/ ml) shows two peaks. Peak 1 (small peak) corresponds to an apparent molecular weight of 120 kDa (hexamer) and peak 2 corresponds to an apparent molecular weight of 44 kDa (dimer).
(0.11 MB TIF)

Figure S4 Western blots of the expression and secretion of EvpC from *E. tarda*. ΔevpC expressing pSA-evpC and N terminus single and double mutants of evpC. The western blot was probed with anti-EvpC rabbit polyclonal antibodies.
(0.07 MB TIF)

Figure S5 Gel filtration (Hiload16/60 Superdex 75 column) profile of EvpC wild type and mutants at 2 mg/ml concentration which shows a single peak around 120 kDa (hexameric EvpC).
(0.05 MB TIF)

Figure S6 Stereo view of the final *2Fo-Fc* electron density map. This map is contoured at a level of 1.0 σ. This figure was prepared using PyMol [41].
(2.55 MB TIF)
