# Supplementary material for: Structural Basis for the Secretion of EvpC: A Key Type VI Secretion System Protein from Edwardsiella tarda
Source: PLoS One. 2010 Sep 23;5(9):e12910. doi: 10.1371/journal.pone.0012910 (PMC2944823; doi:10.1371/journal.pone.0012910)
Supplement: Table S1 — The sequence identity of EvpC with various homologous proteins from selected pathogenic bacterial species. (0.03 MB DOC) [file pone.0012910.s001.doc]

**Supplementary Table 1.**

| Species | Identity (%) | Similarity (%) |
| --- | --- | --- |
| *Photorhabdus luminescens* | 35 | 54 |
| *Geobacter species* | 36 | 56 |
| *Salmonella enterica* | 36 | 53 |
| *Salmonella typhimurium* | 35 | 54 |
| *Enterobacter sakazakii* | 31 | 53 |
| *Azoarcus species* | 33 | 49 |
| *Pseudomonas aeruginosa* | 28 | 47 |
| *Pseudomonas syringae* | 28 | 47 |
| *Pseudomonas putida* | 32 | 51 |
| *Yersinia pestis* | 29 | 49 |
| *Xanthomonas campestris* | 28 | 47 |
| *Burkholderia multivorans* | 29 | 49 |

Identity and similarity of EvpC homologues.
