# Supplementary material for: Structural Basis for the Secretion of EvpC: A Key Type VI Secretion System Protein from Edwardsiella tarda
Source: PLoS One. 2010 Sep 23;5(9):e12910. doi: 10.1371/journal.pone.0012910 (PMC2944823; doi:10.1371/journal.pone.0012910)
Supplement: Figure S1 — Sequence alignment of EvpC and its homologues. The alignment was performed using ClustalW [41], and this figure was prepared using ESPript [42]. (0.02 MB PDF) [file pone.0012910.s002.pdf]

```
                               1        10                   20            30           40            50           60
Sequence                       MAFDTYIKLDK....VDGESTDD..KHKKWIEVLGFAWGAGNEC.TMESGTQGLNTGKAMMSVLRVTKWM
UniRef100_Q6EE19               MAFDTYIKLDK....VDGESTDD..KHKKWIEVLGFAWGAGNEC.TMESGTQGLNTGKAMMSVLRVTKWM
UniRef100_O52405               MAFDTYIKLDK....VDGESTDA..KHKKWIEVLGFAWGAGNEC.SMESGTQGLNTGKATMSVFRITKWM
UniRef100_A4LW48               MAFDAFLKIEG....IPGESTDD..KHKEWIEILSYNFSVMQRPSGSASTSGGASSERATFSDFNIVKAL
UniRef100_Q7N4M6               MSSSIFLQIDG....IKGESTDS..KHKEWIELEHVNFGLFNHARIADSGKRKITVGAADFRTIDCVKVM
UniRef100_Q7NR08               MAFDAFLKIDG....IPGESGDD..KHKDWIEIQSFSHKLEQPAQASASTAGGATAERVNHGVYEITHFL
UniRef100_Q7AXY8               MSYDIFLKIDG....IDGESMDD..KHKNEIEVLSWRWNIHQESTMHAGSGLGSG..KVSVTNLSFEHYI
UniRef100_Q5PFC0               MAYDIFLKIDG....IDGESMDD..KHKNEIEVLGWRWNIHQESTMHAGSGLGSG..KVSVTNLEFEHYI
UniRef100_Q7AXZ0               MAYDIFLKIDG....IDGESMDD..KHKNEIEVLSWRWNIHQESTMHAGSGLGSG..KVSVTNLDFDHYI
UniRef100_A1K517               MAFDAFLRIEG....IPGESTDD..KHQDWIEVLSFNHSMEQPASATASSVGGATAERVNHGSFEVTHLL
UniRef100_A3HIK1               MAFDAYIKIDD....IAGEALDE..KHSKWIEVTGYDFGVSQSTSATASSAGGATSGRAEVSHFTFTKFL
UniRef100_Q5PFB2               MSYDIFLKIDG....IDGESMDD..KHKNEIEVLSWRWNIHQESTMHAGSGLGSG..KVSVTNLSFEHYI
UniRef100_UPI00015DC19E        ..MAIYVKYDG....IDGEATQQ..DHKKWIDVLSLSWGVGRGISTVSGSGNNREASEPSISEVSIVKMF
UniRef100_UPI00015DC19C        .MVAIYVKYDG....IDGEATHQ..DHKKWIDVLSLSWGVGRGISTVSGSGNNREASEPSISEVSIVKMF
UniRef100_A3HMU7               MAFDAYIQIAE....IAGEAKDE..QYSNWIEILGYTFGASQSTSVTASSAGGATSGRTTLSNLTFTKYL
UniRef100_A1FFP6               MAFDAYIQIDT....IPGEALDE..KYKKWIEVIGYDFGVSQSTSATASSAGGATSGRTTVTNFTFRKYL
UniRef100_A3HQ51               MAFDAYIQIDR....IPGEARDE..KYSQWIEVLSYDFGVSQSTSATASSAGGATSGRTSVTNFTFTKYL
UniRef100_Q1QVA0               MAFDAYLKIDG....IPGESLDD..KHPDWIELKSFDFGASQATSATASSSGGASAERVNLSEFRIQKFV
UniRef100_A3HCC2               MAFDAYIQIDG....IPGEVLDE..KHKDWIEVLGYEYGATQATSATASSSGGASSERVALSDFKIRKAV
UniRef100_Q4ZLI0               MAFDAYIQIDG....IPGEVLDD..KHKDWIEVLGYEFGATQATSATASSSGGASSERVALSDFSIRKSV
UniRef100_A1FBX3               MAFDAYIQIDG....IPGEVLDE..KHKDWIEVLGYEYGATQATSATASSSGGASSERVSLTDFKIRKAV
UniRef100_Q48Q84               MAFDAYIQIDG....IPGEVLDD..KHKDWIEVLGYEFGATQATSATASSSGGASSERVALSDFSIRKSV
UniRef100_A4XUR6               MAVDMFIKIGD....LEGESADK..THGKEIDVLAWSWGMSQSGNMHQGGGGAG..KVSIQDISITKWI
UniRef100_Q88IB0               MAFDAYIQIDG....IPGEVLDE..KHKDWIEVLGYEYGATQATSATASSSGGASSERVALSDFKIRKAV
UniRef100_Q88DE1               MAFDAYVHIAE....ITGEALDK..QYANWIEIIGYKFGANQSTSATASSAGGASSGRTTLTNFTFTKYL
UniRef100_Q48KG2               MAFDAFIQIAD....IPGEALDE..KYSKWIEIIGYNFGVSQSTSATASSAGGASSGRTTMTNFTFTKYL
UniRef100_A5W3Q8               MAFDAYIQIDG....IPGEVLDE..KHKDWIEVLGYEYGATQATSATASSSGGASSERVALSDFKIRKAV
UniRef100_Q88CE3               MAFDAYIQIAE....ITGEALDQ..QYANWIEIVGYKFGVSQSTSATASSAGGASSGRTTLTNFTFTKYL
UniRef100_Q48DD5               MAFDAFIQIAD....IPGEALDE..KYSKWIEITGYNFGVNQSTSATASSSGGASSGRTTMTNFTFTKYL
UniRef100_Q3ZV17               MAQDMFIKIDG....IEGESLDA..THKNEIQVLAWHWSVSQHSNMHSGSGGGSG..KATVADFCFEHYV
UniRef100_A1JTM5               MSQDMFIKIDG....IEGESLDA..NHKNEIQVLAWKWDVSQHSNMHSGSGGGSG..KATVSDFCFTHYT
UniRef100_UPI00005F7F4B        MAQDMFIKIDG....IEGESLDA..NHKNEIQVLAWKWDVSQHSNMHSGSGGGSG..KASVSDFCFAHYI
UniRef100_Q4V2U6               MGVAMFMKVDG....VTGESADA..QHKGWTDIQSFSWGASQPGAMASGSGGNAG..KASFNDLVVAAYM
UniRef100_Q668S3               MAQDMFIKIDG....IEGESLDA..NHKNEIQVLAWNWDVAQHSNMHSGSGGGSG..KASVSDFCFAHYI
UniRef100_Q8CLE0               MAQDMFIKIDG....IEGESLDA..NHKNEIQVLAWNWDVAQHSNMHSGSGGGSG..KASVSDFCFAHYI
UniRef100_UPI00015DB91F        ..MAIYVNYDG....IQGEATQQ..DHKKWIDVLSLSWGVGRGISTVAGSGNNREASEPSVSEVSIVKAF
UniRef100_Q4LBF6               MAFDGYIQIAE....IPGEALDE..KFSKWIEITGYNFGVSQSTSATASSSGGASSGRTTMTNFTFTKYL
UniRef100_A0FY79               MAQDIFLKIDG....IDGESQDD..KHKNEIEVLNWNWEISQESSMHSGSGGGAG..KATVGDLWFQHGI
UniRef100_UPI0000558E48        ........MDG....VTGESADA..QHKGWTDIQSFSWGASQPGAMASGSGGNAG..KASFNDLVVAAYM
UniRef100_A0UFU8               MAQDIFLKIDG....INGESLDD..KHKDEIEILNWDWEILQESSMHSGSGGGAG..KATVKDLTFEHNI
UniRef100_Q398L5               MSQDIFLKIDG....INGESLDD..KHKDEIEILNWDWEIQQESSMHSGSGGGAG..KATVKDLTFEHNI
UniRef100_Q2T8N7               MGVAMFMKVDG....VTGESADA..QHKGWTDIQSFSWGASQPGAMASGSGGNAG..KASFNDLVVAAYM
UniRef100_A4BPJ1               .MLDCFLVIDE....IKGESKDS..KYPDAVEVLAWSWGASQQGTMHFGGGGAG..KASFQDISLTKYI
UniRef100_Q02CH0               AQVDYFLKIDG....IDGESLQK..GHEKEIEIMSFSWGESNAGSFAGNLGGGSG..KVSMQDFHFTVPV
UniRef100_Q4ZP53               MAFDAYIKIEG....IPGEALDD..RYKDCIEITGYGFGMHQSTSATASSSGGASSGRTSLSDFTFTKSL
UniRef100_Q3JHV3               MAQDIFLKIDG....INGESLDD..SHKDEIEVLNWNWEIQQESTMHTGSGGGAG..KASVKDLTFEHAI
UniRef100_Q396B1               MGVAMFMKVDG....VTGESADA..QHKGWTDIQSFTWGASQPGAMASGSGGNAG..KASFNDLVVAAYM
UniRef100_A3NG67               MAQDIFLKIDG....INGESLDD..SHKDEIEVLNWNWEIQQESTMHTGSGGGAG..KASVKDLTFEHAI
UniRef100_Q9I747               MAVDMFIKIGD....VKGESKDK..THAEEIDVLAWSWGMSQSGSMHMGGGGAG..KVNVQDLSFTKYI
UniRef100_A6UXL8               MAVDMFIKIGD....VKGESKDK..THAEEIDVLAWSWGMSQSGSMHMGGGGAG..KVNVQDLSFTKYI
UniRef100_Q500J7               MAFDAYLKIDG....IPGEALDA..QFKDWIEMENFDLGASQSASVTATSAGGATSGRARMSDLFFRKTV
UniRef100_Q0B9Z3               MAQDIFLKIDG....INGESLDE..KHKDEIEVLTWEWDIQQESTMHAGSGGGAG..KATVKDLTFEHNI
UniRef100_A0Z107               MAVDAFLKLDG....IDGESQDK..EHKGEIDIISWSWGGVSTGSFDLGGGASG..KASFQDMMLTKYY
UniRef100_A0TH61               MAHDIFLKING....IDGESKDA..SHQNEIEILSWSWNVSQQSNMHLGSGGGAG..KATVDDLQFEHYI
UniRef100_Q48C23               MAFDAYLKIDG....IPGEALDA..QFKDWIEMENFDLGASQSASVTATSAGGATSGRARMSDLFFRKTV
UniRef100_A3Q9C1               AATDMFLKIDG....VDGESLDA..QHRNEIDVLAWSWGASS..DMR.........RTCIQDISVTKYV
UniRef100_Q89P91               MAVDIFLKLDP....IKGESLDD..KHKDEIDILSWSFGESQSGTFHSGSGGGAG..KVSVQDLHFTHFV
UniRef100_Q4K3N6               MAVDIFIKIGD....IKGESMDK..AHKDEIDVLNWSWGMSQSGNMHVGGGGAG..KVNIQDLSLTKYV
UniRef100_A8GG66               MAIDMFLKVEG....ASGESKDS..NHKGWTDITSFSWGASQPGNMSVGGGGAG..KVCFNDLHVNALV
UniRef100_Q4IYV8               MSTSMHLKIEG....VQGESQES..QHAAWLDIKSFTWGISQPGSLDSGGGTPG..RASFTDLHVVSYV
UniRef100_A6GLL0               ....MFLKIKG....VDGESVDS..VHAKEIDIAAWSWGMSQSGTTHVGRGGAG..KVSVQDISFTKYI
UniRef100_A2W6K2               MAHDIFLKING....IDGESRDA..THQNEIEVLSWSWNVSQQSNMHLGSGGGAG..KATVDDLQFEHYI
UniRef100_Q0KDW9               MAQDIFLKING....IDGESQDS..SHKNEVEVLAWDWSIEQQSTMHAGSGGGAG..KATVSDLSFEHFI
UniRef100_A0TLV3               MAHDIFLKING....IDGESKDA..THHDEIEVLSWSWSVSQQSNMHLGSGGGAG..RATVDDLQFEHYI
UniRef100_A7MQ13               MAIDMFLKVDG....VTGESKDS..NHTGWTDITSFSWGASQPGNMSVGGGGAG..KVNFNDLHVNALI
UniRef100_Q4ZV44               MSFDAFMQVDG....VEGESLDD..AHKGWVELLSYHYDAVQSVSTTACSSGGATAGGVTLGDLRVSKYI
UniRef100_Q3BMP1               MAFDMHIKFGSGKVKIEGASNHK..KHKGEIPILAWSWGASNSGDLHTGGGSASGG.KAHIQDISITKYV
UniRef100_A3P1G9               MSHDIFLKING....IDGEAEDA..THKSEIEVLSWSWNVSQQSNMHLGSGGGAG..KATIDDLQFEHYI
UniRef100_Q8PF41               MAFDMHIKFGSGKVKIEGASNHK..KHKGEIPILAWSWGASNSGDLHTGGGSASGG.KAHVQDISVTKYV
UniRef100_Q63P47               MSHDIFLKING....IDGEAEDA..THKGEIEVLSWSWNVSQQSNMHLGSGGGAG..KATIDDLQFEHYI
UniRef100_Q48C28               MSFDAFMQVDG....VEGESLDD..AHKGWVELLSYHYDAVQSISTSASSSGGASAGGVTLGDFKVSKYI
UniRef100_Q21KI0               ..MSIFVNYEG....IKGESSDS..GHKGWMDVEAVSWGVGRNITSNSSTQGDRESSNAVISDLHVTRYM
UniRef100_A4LM29               MSHDIFLKING....IDGEAEDA..THKGEIEVLSWSWNVSQQSNMHLGSGGGAG..KATIDDLQFEHYI
UniRef100_A3NFP4               MSHDIFLKING....IDGEAEDA..THKGEIEVLSWSWNVSQQSNMHLGSGGGAG..KATIDDLQFEHYI
UniRef100_A3KB44               ......MKLGNG...IKGESQND..QHKDWIDVLSWSVGMTQSGTTHLGSGGGGG..KVDVGDIAFTKYV
UniRef100_A7CMP7               MAQDIFLKING....IDGESQDS..SHKNEIEVLVWDWNIEQQSNMHAGSGGGAG..KATVSDLSFEHYV
UniRef100_A1TM68               MSVDMFMKVEG....ASGESQDA..NHKSWTDIRSFTWGATQPNSMATGSGAGAG..KVTFHDLNVVAKI
UniRef100_A8GG80               MAIDSFLKVDG....VTGESNDS..NHKNWTDILSFNWGATQSGNMAVGGGGTG..KVNFKDLEVQALI
UniRef100_Q2T924               MSHDIFLKING....IDGEAEDA..THKGEIEVLSWSWNVSQQSNMHLGSGGGAG..KATVDDLLFEHYI
UniRef100_A0NQ25               MAVDFYLELDG....IKGEAQDK..THKDKIDVLSWSWGASQSGTTHMGSGSGSG..KAHFQDLSITKFV
UniRef100_A0GAP0               MAQDTFIKING....CDGESQDA..SHLNEIDVIGWRWKISQDSTMMAGSGGGAG..KASVSDLEFTHKL
UniRef100_Q7W1H3               MAVDMFMKIEG....ANGESKDA..NHKDWTDIVSFSWGATQPASLATGGGQGVG..KASFNDLHVVARI
UniRef100_Q3BTR0               MAFDMHLKFGSGDVEIKGASNHS..KHKDEVPIIAWSWGTSNTGNLHTGAGYAAGG.KANVQDISVTKYV
UniRef100_Q13YG2               MAQDIFLKING....IDGESPDA..AHKNEIEVKSWGWRVSQQANMHAGSGGGAG..KATVDDLEFEHLV
UniRef100_Q48Q45               MSFDAFMKVDG....VEGESLDD..GHRGWIELLSYHYDAMQSISQTASSSGGATAGGVSLGDFQISKYV
UniRef100_Q2P1B8               MAFDMHLKFGSGDVEIKGASNHT..KHKDQVPIIAWSWGTSNTGNLHTGAGYAAGG.KANVKDISITKYV
UniRef100_Q3IWK2               MAFTGYLKIDD....IKGESRRA..DHEDEIDAFGVSWLVEQTSSASTGSG..RTRGRARVGDFRLRKWM
UniRef100_A1KCF7               MAVDMFIKIGD....IKGESVDK..VHADKIDVLAWSWGMSQSGTTHMGSGGGSG..KVSVQDISFTKYV
UniRef100_Q48E46               MSLDAYLQIEG....IPGETLSE..GYENWIELQDFDLSASQTASATSTSAGGATSGRAYMSDMLVRKAV
UniRef100_A7MRP5               MAIDMFLKVEG....VTGESKDA..NHASWIDVLSFNWGAAQPGNMAVGGGGAG..KVNFQDLAVQAIV
UniRef100_A3PPF7               MAFTGYLKIED....IKGESRRA..DHEDEIDAFGVSWLVEQTSSASTGSG..RTRGRARVGDFRLRKWM
UniRef100_Q0FRC9               MAFDAYMYFPGQS.RVQGETHDEAMAAKKAFEIVSFNFGAENNI.NIGSATGGGGAGKAWFKDFEVAKST
UniRef100_Q0FN14               MAFTGYLKIPD....IDGESQRA..EHEEEIDITDIMWSVEQAATAQTGRG..RARSRAEVSSLYCKKVY
UniRef100_Q7WP83               ......MKIEG....ANGESKDA..NHKDWTDIVSFSWGATQPASLATGGGQGAG..KASFNDLHVVARI
UniRef100_Q6F922               .MKDIYVQFRGKY.KVDGESRDS..EHKDWLEVNSWSHNIRQPKSATSSSVGGHTAERVEHSDMLFVKDL
UniRef100_Q13MH0               MVQDTFIKIGG....IDGESQDV..SHLNEIDVIGWRWKVSQQAAMMSGSGGGAG..KATVSDLEFTHQL
UniRef100_A3TYB1               MAFDAFLYFPNDS.KMVGETQDKSMKENKATEILSFEIGAENNI.NIGSITAGGGAGKATFKELTITKKT
UniRef100_Q2P080               .MQHAFLSID....TIKGESHDD..KHTDWVEIKSFSQDLLQPRSATASTAGGQSAARVNLSPIEIVKSI
UniRef100_Q28MX3               ..MPIYMQLDG....IPGDATHE..THRNWMDIETLHWNVSRNMNTQAGSTANREASEPTVSEVVLTKVT
UniRef100_A0VH35               MSVDMFMKVEG....ASGESQDS..NHKGWTDIDSFTWGATQPNTMSSGGGAGAG..KVSFHDLNVIAKV
UniRef100_A0GJN3               MAQDIFIKING....IDGESQDS..AHKNEIEVASWGWQILQQSNMHMGSGGGAG..KATVEDLAFEHLV
```

```
                                  70        80        90             100         110
                                  .         .         .              .           .
Sequence                      DCASVKLASAAVQGQNFPTLELEICTQ....AG.DKFAFCIYKFTH.VAVSSYQCSG...........A.
UniRef100_Q6EE19              DCASVKLASAAVQGQNFPTLELEICTQ....AG.DKFAFCIYKFTH.VAVSSYQCSG...........A.
UniRef100_O52405              DCASVKLASAAVQGQNFPSLEVEICTQ....AG.DKFAFCIYKFTN.VAISSYDCSG...........A.
UniRef100_A4LW48              DKASPKLFEACATGRHIPTITLELCRAGGD....KLKYMEYKLSN.VIISMNRPGGT.........S.
UniRef100_Q7N4M6              DSSSIGLLSAVAQGTAIKKVKLEICTIF...KG.EMHPYAEVEMDE.CIISDVHETC..........S.
UniRef100_Q7NR08              DKASPKIYEACCTGKHIKEITIELCRAGGD.....KVKYMEIKMEQ.VLISKVEPHG..........S.
UniRef100_Q7AXY8              DRASPNLFKYCSSGKHIPQAILVMRKAGGN.....PLEYLKYTFTD.LIIAMVSPSGS...........
UniRef100_Q5PFC0              DRASPNLFKYCASGKHIPQAILVMRKAGGN.....PLEYLKYTFTD.LIVAVVSPSGN...........
UniRef100_Q7AXZ0              DRASPNLFKYCASGKHIPQAILVMRKAGGN.....PLEYLKYTFTD.LIVAVVSPSGS...........
UniRef100_A1K517              DQASPKIYDACCTGKHIKEVTLELCRAGGD.....KVKYMEVKLEQ.VVISKVVPAG...........A.
UniRef100_A3HIK1              DSASCKLMEASCAGQHLKEVKLALCRAGGD.....KLKYYEIILEE.VIIADYTQS.............
UniRef100_Q5PFB2              DRASPNLFKYCSSGKHIPQAIPVMRKAGGN.....PLEYLKYTFTD.LIIAMVSPSGS...........
UniRef100_UPI00015DC19E       DASSPKLFTEACTGNTGKTVKIDLTTTGSP.....GATYCTYTLTN.ALISSYSVS............SG
UniRef100_UPI00015DC19C       DASSPKLFTEACTGNTGKTVKIDLTTTGSP.....GATYCTYTLTN.ALISSYSVS............SG
UniRef100_A3HMU7              DNASCKLLEASCAGQHLKEVKLVVCRAGSD.....KLKYYEVVLEE.VIIADYAQS.............
UniRef100_A1FFP6              DSASCKLMEASCTGQHLKEVKLALCRAGGD.....KLKYYEIILEE.VIIAEYSQC.............
UniRef100_A3HQ51              DSASCKLMEASCTGQHLKAVKLALCRAGGD.....KLKYYEIILEE.VIIAEYSQS.............
UniRef100_Q1QVA0              DKASAKLFESCCKGQHIKEVVLHVNRAGGD.....KVRYMEIKLEE.VIVSSTEMEGTSTATGFEQGEN.
UniRef100_A3HCC2              DKASAKLFEHCCTGKHIAKLKLNVNRAGGD.....KVTYLTIDMEE.VVVSSVKFI....AGGNQGGED.
UniRef100_Q4ZLI0              DKASAKLFEACCKGTHIAKIQLNVNRAGGD.....KLTYLTINLEE.VVVSSVKAV....AGGSQKGED.
UniRef100_A1FBX3              DKASAKLFEHCCTGKHIAKLKLNVNRAGGD.....KVTYLTIDMEE.VVVSSVKFV....AGGNQGGED.
UniRef100_Q48Q84              DKASAKLFEACCKGTHIAKIQLNVNRAGGD.....KLTYLTINLEE.VVVSSVKAV....AGGNQKGED.
UniRef100_A4XUR6              DKSSPNLMMACSNGKHFPEAKLTVRKAGGES....PVEYLIITLKE.VLVSSVSTGGS...........
UniRef100_Q88IB0              DKASAKLFEHCCTGKHIAKLKLNVNRAGGD.....KVTYLTIDMEE.VVVSSVKFV....AGGNQSGED.
UniRef100_Q88DE1              DSASCKLLEASCAGQHLKEVKLVLCRAGGD.....KLKYYEVVLEE.VIIADYAQS.............
UniRef100_Q48KG2              DSASCKLMEASCAGQHLKEVKLVLCRAGTD.....KLKYYEVVLEE.VIIADYTQS.............
UniRef100_A5W3Q8              DKASAKLFEHCCTGKHIAKLKLNVNRAGGD.....KVTYLTIDMEE.VVVSSVKFV....AGGNQNGED.
UniRef100_Q88CE3              DSASCKLLEASCAGQHLKEVKLVVCRAGTD.....KLKYYEVVLEE.VIIADYAQS.............
UniRef100_Q48DD5              DSASCKLMEASCAGQHLKEVKLVLCRAGTD.....KLKYYEVVLEE.VIIADYTQS.............
UniRef100_Q3ZV17              DKATPNLVSYCLTGKHIKNIQFVIRKAGGD.....PLEYLLIKFTD.VIITGVDVMGS...........
UniRef100_A1JTM5              DKASPNLLSYCLLGKHIKNIQFVLRKAGGD.....PLEYLTIKFTD.VIITRVDMAGS...........
UniRef100_UPI00005F7F4B       DKASPNLLSYCLLGKHIKNIQFVLRKAGGD.....PLEYLTIKFTD.VIITRVDMAGS...........
UniRef100_Q4V2U6              DKGATAIIKNCASGKHLPTVEISACKTG.GS....QIEFMRVTLQE.VLVTSAQIAGVD..........P
UniRef100_Q668S3              DKASPNLLSYCLLGKHIKNVQFVLRKAGGD.....PLEYLTIKFTD.VIITRVDMAGS...........
UniRef100_Q8CLE0              DKASPNLLSYCLLGKHIKNVQFVLRKAGGD.....PLEYLTIKFTD.VIITRVDMAGS...........
UniRef100_UPI00015DB91F       DAASPKLFTESCTGNVGKTVKIDLVSTGNP.....GVTYCTYTLTN.ALISSYSVS............TG
UniRef100_Q4LBF6              DSASCKLMEASCAGQHLKEVKLVLCRASTD.....RLKYYEVVLEE.VIIADYSQS.............
UniRef100_A0FY79              DRASPNLMKYALTGKHIDQAVLVMRKAGGN.....PLEYLKITMSD.VLVTRVKPSGS...........
UniRef100_UPI0000558E48       DKGATAIIKNCASGKHLPTVEISACKTG.GS....QIEFMRVTLQE.VLVTSAQIAGVD..........P
UniRef100_A0UFU8              DRASPNLMKYALTGKHIDQAVLVMRKAGGN.....PLEYLKLTMSD.VIVTRVKPSGS...........
UniRef100_Q398L5              DRASPNLMKYALTGKHIDQAVLVMRKAGGN.....PLEYLKLTMSD.VIVTRVKPSGS...........
UniRef100_Q2T8N7              DKGATAIIKNCANGKHLSSVEISACKTG.GS....QIEFMRVTLQE.VLVTSAQIAGVD..........P
UniRef100_A4BPJ1              DKSSTALWKFLTNGKHFEKGKLVIRKAGGE.....QLEYLVLEMSK.ILVSSISTGGS...........
UniRef100_Q02CH0              NKSSPKLFLACATGEHIKSAVLVCRKAG.KT....QQEFLKWKFTD.LLISSYNTGGS...........
UniRef100_Q4ZP53              DKSSCKLMESLCAGKHLKEVVLSLHRAGGD.....KVKYFEVLLEE.VIISNYTQH.............
UniRef100_Q3JHV3              DRASPNLMKYALTGKHVDQAVLVMRKAGGN.....PLEYLKLTMSD.VIITRVRPSGS...........
UniRef100_Q396B1              DKGAPAIIKNCANGKHLSTVEISACKTG.GT....QVEFMRVTLQE.VLVTSAQVAGID..........P
UniRef100_A3NG67              DRASPNLMKYALTGKHVDQAVLVMRKAGGN.....PLEYLKLTMSD.VIITRVRPSGS...........
UniRef100_Q9I747              DKSTPNLMMACSSGKHYPQAKLTIRKAGGEN....QVEYLIITLKE.VLVSSVSTGGS...........
UniRef100_A6UXL8              DKSTPNLMMACSSGKHYPQAKLTIRKAGGEN....QVEYLIITLKE.VLVSSVSTGGS...........
UniRef100_Q500J7              DKATPKLHEACCSGKHFKEVTLAVNRAGTD.....KLKYLEIVLEE.VIISSVSLN..........GNG.
UniRef100_Q0B9Z3              DRASPNLMKYALTGKHIDQATLVMRKAGGN.....PLEYLKLTMSD.VIVTRVKPSGS...........
UniRef100_A0Z107              DKASPNLMSKLADGTHIKEATLFARKAGGSS....AIEFLKIKLTD.VIITSVGLGGS...........
UniRef100_A0TH61              DRASPNLIQYCLLGKHIDEAKLTVRKAGGN.....PLEYILLTMND.VLVTQVSPSGV...........
UniRef100_Q48C23              DKATPKLHEACCSGKHFKEVTLAVNRAGTD.....KLKYLEIVLEE.VIISSVSLN..........GNG.
UniRef100_A3Q9C1              DSASPQLLMNQMSHMQYPRATLTVRKAGGT.....PLEYIVIELRD.VWVSSLSTGGS...........
UniRef100_Q89P91              DKATPELFKVCANGKHIDTAILTVRKAG.EN....PLEYLKIEMKH.ILVTSVSTGGS...........
UniRef100_Q4K3N6              DKASPNLMMHCSSGKHIDKVRLTVRKAGGES....QVEYMIIDLEE.VLITSLSTGGS...........
UniRef100_A8GG66              DKSTPALLKHCSSGKHLTKIELSVCKAG.GS....QVEYVKITLDD.VLVTAVQYTGAG..........
UniRef100_Q4IYV8              EASAPTAFKKCANGTSLGTVELSCSRLSDGD....KMVFMTITLTN.VLVTKADIKGVD..........R
UniRef100_A6GLL0              DKATPNLIKACCNGKHFDEAVLTVRKAGEK.....PLEYVVLTMKD.VIISNVSQGGS...........
UniRef100_A2W6K2              DRSSPNLVQYCLLGKHIDEAKLTVRKAGGN.....PLEYILLTMND.VLVTQVSPAGV...........
UniRef100_Q0KDW9              DRASPNLMKYCLTGKHINEAVLVVRKAGGN.....PLEYLKLTMTD.VIVTKVSPKGS...........
UniRef100_A0TLV3              DRASPNLIQYCLLGKHIDQAKLTVRKAGGN.....PLEYILLTMND.VLVTHVSPAGV...........
UniRef100_A7MQ13              DKSTTAILKHCSSGKHLTKVELSVCKAG.GQ....QVEYTRITLED.VLVTSVQYTGAD..........
UniRef100_Q4ZV44              DKATPKLFELCCRGSHIKKVTIRVHRAGTE.....KFKYLDIVLEE.VLISTISGN............G.
UniRef100_Q3BMP1              DGCSNALLDAVCTGARVENAWLYVTNATGE.....QSDFLTLELSEGVLITSLSTGGS...........
UniRef100_A3P1G9              DRASPNLVQYCLLGKHIDEARLVVRKAGGS.....PLEYIKLTMSD.VLVTQVSPAGV...........
UniRef100_Q8PF41              DGCSNALLDAVCTGARVENAWLYVTNATGE.....QSDFLTLELSEGVLITSLSTGGS...........
UniRef100_Q63P47              DRASPNLVQYCLLGKHIDEARLVVRKAGGS.....PLEYIKLTMSD.VLVTQVSPTGV...........
UniRef100_Q48C28              DRATAKLFELCCRGSHIKKVTIRVHRAGTE.....KFKYLDIVLEE.VLISTVSGN............G.
UniRef100_Q21KI0              DSSTPKLFIESCCG.TGKEVKIHLSKTGSGS...GADVYMEYTLKN.ALISSYSVN............AV
UniRef100_A4LM29              DRASPNLVQYCLLGKHIDEARLVVRKAGGS.....PLEYIKLTMSD.VLVTQVSPAGV...........
UniRef100_A3NFP4              DRASPNLVQYCLLGKHIDEARLVVRKAGGS.....PLEYIKLTMSD.VLVTQVSPAGV...........
UniRef100_A3KB44              DAASHDLIKRCCSGEHIDTAELHVLKAGGSK....PVDYMKIKMKD.VMITSYNTGGMN..........
UniRef100_A7CMP7              DRASPNLMKYCLTGKHIDQATLVVRKAGGN.....PLEYLKLTMSD.VIVTRVAPRGA...........
UniRef100_A1TM68              DKAYPAVLKHCSSGKHLGQVEISMCKAG.GT....QIEFSKITLTD.VLVTSVTVSGTD..........
UniRef100_A8GG80              DKSTPAILKFCSSGKHVTKVELSICKAG.GT....QVEYTRIVLED.VLVSMADFTGVG..........
UniRef100_Q2T924              DRASPNLVQYCLLGKHIDEARLVVRKAGGS.....PLEYIKLTMND.VLVTQVSPAGV...........
UniRef100_A0NQ25              DKSTPVLLQHVSTGKHITKGTLFVRKAAGDV....PLEYIKLEMKD.IIVTNVSTGGS...........
UniRef100_A0GAP0              DRASPNLAKYCFTGKHVDQVKLTVRKAGGM.....PFEYLKITMYD.VIITQVEPIG............
UniRef100_Q7W1H3              DKAAPAVMKHCASGKHLGKIELSVCKAG.GA....QVEYSKVTLED.VLVTSVQIAADN..........
UniRef100_Q3BTR0              DSCSNALLNACCTGARVDSATLYVTNATGE.....QTDYVTIELSEGVMITSVSTGGS...........
UniRef100_Q13YG2              DRASPNLMKYCLTGKHVDQAVLVVRKAGGN.....PLEFLKITMSD.VIVTQVHPSGS...........
UniRef100_Q48Q45              DRATPKLFELCCKGSHIKNVTIRIHRAGTE.....KFKYLDIVLEE.VLIALVSGQ............G.
UniRef100_Q2P1B8              DSCSNALLNACCTGARVDSATLYVTNATGE.....QSDYVTIELSEGVMITSVSTGGS...........
UniRef100_Q3IWK2              DASSPYLALACMQGKSFPEMIFMARKDSGE....AHLDYLQVTLKN.CIVSSYAL.........AQDAPE
UniRef100_A1KCF7              DASSHALIMACCNGKHYDEAVLIVRKAGEK.....PLEYIKVTMKE.VIVTSVSTGGS...........
UniRef100_Q48E46              DNATPKLHEACCSGKHFKQVSVHLFRAGDP.....KVKYLEIKLEE.VIISSFSLT..........GNG.
UniRef100_A7MRP5              DKGTPAILKFCASGKHVNKVELSVCKAG.GQ....QVEYSKIVLED.VLVTRTEFTGVG..........
UniRef100_A3PPF7              DASSPYLALACMQGKSFPEMIFMARKDSGE....AHLDYLQVTLKN.CIVSSYAL.........AQDAPE
UniRef100_Q0FRC9              DTASCGLFSTLCNGTHFDEAIIELRRSGGSASA.SGSTFMKFHFKL.VMVKDMTWEG...........S.
UniRef100_Q0FN14              DASSPYIALAAMQAKSFKDVIVTVRRDSGD....AHLDYLTITMTN.TIISRYEVNGVG...RITDSEGK
UniRef100_Q7WP83              DKAAPAVMKHCASGKHLGKIELSVCKAG.GA....QVEYSKVTLED.VLVTSVQIAADN..........
UniRef100_Q6F922              DATSPKLWEACSAGYTFDEVQIDFYRANGD....KRIKYLQIKLKH.VLVSSVTPT.............
UniRef100_Q13MH0              DRASPNLTRYCFTGKHIDQAKLTVRKAGGL.....PFEYLKITMYD.VVITQVEPIG............
UniRef100_A3TYB1              DLMSDMMFESCVTGAHFDTMIIELRRSAQGGGG.KAGTFLMWEFKL.VMIQDISWSG...........S.
UniRef100_Q2P080              DLSTTALNQACANGTTFPKARIEFMRADKD...GNAINYYLVELMN.VLVHRVTTT.............
UniRef100_Q28MX3              DSSSTRLFAEACTGRTGKLVTIHLVTTGNP.....GDTYVEYKLTN.TLVSNYSVD............TS
UniRef100_A0VH35              DKCYPAVLKHCATGKHLGQVDISLCKAG.GT....QIEFTKITLTD.VMVTNVTVSGAN..........
UniRef100_A0GJN3              DRASPNLMKYCLTGKHIDQAVLTVRKAGGN.....PLEYLKIMND.VIVTQVHPSGS...........
```

```
                        120       130       140            150       160
Sequence                TGGSDRPQETIDFAYKEVTWEYVPQDQNGK...AGGKIGPEGWSLITNKKK
UniRef100_Q6EE19        TGGSDRPQETIDFAYKEVTWEYVPQDQNGK...AGGKIGPEGWSLITNKKK
UniRef100_O52405        SGGSDRPQETIDFAYKEVTWEYIPQDQSGK...AGSKIGPEGWSLITNQKK
UniRef100_A4LW48        HADETLPLEEISFNYGKIELAYTHQNRADG...SSGGQIAAGWNLESNKK.
UniRef100_Q7N4M6        RGG.EFPQEQISIAYSKIKWTFTPLKEDGT...KGTKVGPEGWDLIENKKQ
UniRef100_Q7NR08        ATDSGFPAEKVSFSYGKIKWTYTQQKRADG...AGGGNVSAGWDLTANKV.
UniRef100_Q7AXY8        QGGEIASRESIELSFSTVKQEYVVQNQQG....GSGGTITAGYDFKANKEI
UniRef100_Q5PFC0        REGEIASRERIELSFSTVKQEYVVQNQQG....GSGGTITAGYDFKANKEI
UniRef100_Q7AXZ0        HDGEIASRETVELSFSTVKQEYVVQNQQG....GSGGTITAGYDFKANKEI
UniRef100_A1K517        ANDQGFPQEKISFSYGRIKWTYTRQKRDDG...TGGGNVSAGWDLTANKA.
UniRef100_A3HIK1        .AADGVPVEVVRLDYGRIKTTYTLQKRIDG...SGGGNIAGGWDRIGNKK.
UniRef100_Q5PFB2        QGGEIASRESIELSFSTVKQEYVVQNQQG....GSGGTITAGYDFKANKEI
UniRef100_UPI00015DC19E GD...RPTESISISFTKLEFKFTPYDDKNK....AGTPVTVSYDLATTKSS
UniRef100_UPI00015DC19C GD...RPTESISISFTKLEFKFTPYDDKNK....AGTPVTVSYDLATTKSA
UniRef100_A3HMU7        .AHAGVPTEIVQLNYGRIKTTYTRQQRVDG...SAGGNVTGGWDRIANKK.
UniRef100_A1FFP6        .VDTGVPLEVVQLNYGRIKTTYTQQRRLDG...AGGGNVAGGWDRIGNKK.
UniRef100_A3HQ51        .VDTGVPLEVVQLNYGRIKTTYTQQRRIDG...GGGGNIAGGWDRIGNKK.
UniRef100_Q1QVA0        EDRHDLPFETVKFNYARIKITYTQQKRSDG...QGGGNVAGGWDRTRNKV.
UniRef100_A3HCC2        KAVSDLPVEEISFNFARIKTTYTQQNRTDG...QAGGNIVGGWDRTANKV.
UniRef100_Q4ZLI0        NAVSDLPIEEISFNFARIKTTYTQQSRSDG...QGGGNVTGGWDRTANKV.
UniRef100_A1FBX3        KAVSDLPIEEISFNFARIKTTYTQQNRTDG...QAGGNIVGGWDRTANKV.
UniRef100_Q48Q84        NAVSDLPIEEISFNFARIKTTYTQQSRSDG...QGGGNVTGGWDRTANKV.
UniRef100_A4XUR6        GGEDRL.TENVSLNFAQVLVDYQPQKADGA...KDGGPVKYGWNIRENVKV
UniRef100_Q88IB0        KAVSDLPVEEISFNFARIKTTYTQQNRTDG...QAGGNIVGGWDRTANKV.
UniRef100_Q88DE1        .ASAGVPVEVVQLNYGRIKTIYTRQKRLDG...SAGGNVTGGWDRINNKK.
UniRef100_Q48KG2        .ASSGIPIEIVQLNYGRIKTTYTLQKRVDG...TAGGNVAGGWDRINNKK.
UniRef100_A5W3Q8        KAVSDLPVEEISFNFARIKTTYTQQNRTDG...QAGGNIVGGWDRTANKV.
UniRef100_Q88CE3        .ASSGVPMEVVQLNYGRINTTYTRQKRLDG...SAGGNVTGGWDRINNKK.
UniRef100_Q48DD5        .ASAGIPIEIVQLNYGRIKTTYTLQKRVDG...TAGGNVAGGWDRINNKK.
UniRef100_Q3ZV17        LEDETRPRESVRFSFTKMTEDYVMQNAEG....QKAGVISASYDVKANLHG
UniRef100_A1JTM5        LEDETRPREEIRFSFTKMTQDYVMQNAEG....HKSGVISANYDVKANQQS
UniRef100_UPI00005F7F4B LEDETRPREEVRFSFTKMTQDYVMQNAEG....HKSGVISANYDVKANLQS
UniRef100_Q4V2U6        GDAADRLMMQYGFQAAKVKKQYWQQNDNG....GKGAEVSVGWNIKENTEM
UniRef100_Q668S3        LEDETRPREEIRFSFTKMTQDYVMQNAEG....HKSGVISANYDVKANMHS
UniRef100_Q8CLE0        LEDETRPREEIRFSFTKMTQDYVMQNAEG....HKSGVISANYDVKANMHS
UniRef100_UPI00015DB91F GD...RPTESVSISFTKLEFKFTPYDDKNK....AGTPVTVSYDLATTKSS
UniRef100_Q4LBF6        .ASSGVPIEIVQLNYGRIKATYTLQKRSDG...AAGGNVTGGWDRIGNKI.
UniRef100_A0FY79        KNGEEKSLEEVSLSFAKVKQEYVVQNAQG....GSGGAVTASYDIKGNKEA
UniRef100_UPI0000558E48 GDAADRLMMQYGFQAAKVKKQYWQQNDNG....GKGAEVSVGWNIKENTEM
UniRef100_A0UFU8        KAGEEKSRETVSLSFSKVKQEYVVQNAQG....GSGGAVTASFDIKGNKEA
UniRef100_Q398L5        KAGEEKSRETVSLSFSKVKQEYVVQNAQG....GSGGAVTASFDIKGNKEA
UniRef100_Q2T8N7        GDAADRLMMQYGFQAAKVKKQYWQQNDNG....GKGAEVSVGWNIKENTEM
UniRef100_A4BPJ1        GGEDRL.TENVSLNFAEFKITYTPQKDDG....GPDAATDFGWSIEKNAAK
UniRef100_Q02CH0        GGSDVVPIDQVSFNFTKIEVSYAPQKQDGS...LD.AALPAGYDLKAGQKV
UniRef100_Q4ZP53        .ANDGIPSETISLNYGKIKTTYTQQKRSDG...AGGGNVSGGWNRINNKV.
UniRef100_Q3JHV3        RDDTERSRETVSLSFAKVKQEYVVQNAQG....GSGGAVTTSFDIKGNKET
UniRef100_Q396B1        GDVADRLMMHYGFQAAKVKKQYWQQNDNG....GKGAEVTVGWNIKENTEM
UniRef100_A3NG67        RDDTERSRETVSLSFAKVKQEYVVQNAQG....GSGGAVTTSFDIKGNKEA
UniRef100_Q9I747        GGEDRL.TENVTLNFAQVQVDYQPQKADGA...KDGGPVKYGWNIRQNVQA
UniRef100_A6UXL8        GGEDRL.TENVTLNFAQVQVDYQPQKADGA...KDGGPIKYGWNIRQNVQA
UniRef100_Q500J7        GVEGGFPTETVRLNYGRIKMTYTQQKRADG...QGGGQVVGGWDAISNKV.
UniRef100_Q0B9Z3        KNDAEKSRESVSLSFAKVKQEYVVQNAQG....GSGGAVTASFDIKGNKEA
UniRef100_A0Z107        GGDDRY.VENVSLAYAKFEVAYQAQKNDGA...KDGGEIKAGWNVQTNESA
UniRef100_A0TH61        AADESRPHESVRLSFSRLKQEYVVQNAQG....GGGGAITATFDIKKNAA.
UniRef100_Q48C23        DVEGGFPTETVRLNYGRIKMTYTQQKRADG...QGGGQVVGGWDAISNKV.
UniRef100_A3Q9C1        GGEDRL.TENITLNFEELKYQYTPQKADG....SGGPAISATITSEKCR..
UniRef100_Q89P91        HGEDRL.TENVSLNFAKVKMTYKTQTEKG....GAGAAPVFGWDVPANKEW
UniRef100_Q4K3N6        GSDDRL.TENITLNFAQVMVEYQPQKADGT...KDGGPIKYGWNIRSNTKR
UniRef100_A8GG66        ..GEDTVGVTYSFQASKVKQQYWEQSDKG....GKGAESSAGWNIKENREA
UniRef100_Q4IYV8        DDEQERLLIEYAFQAPEVEFGYTPQQDDG....SAGGEVTMGWNIPQNTEL
UniRef100_A6GLL0        GGEDRL.TETVTLNFAEFSYVYVPQKKDGG...PEGK.VEATFNIATNSEK
UniRef100_A2W6K2        ADDESRPREAVRLSFSRFKQEYVVQNAQG....GSGGAITATFDIKRNAA.
UniRef100_Q0KDW9        VDDEVRMREKVALSFSRVKQEYVVQNAQG....GSGGAVTAGYDIKGNKEA
UniRef100_A0TLV3        AADESRPREIVRLSFSRLKQEYVVQNAQG....GSGGAITATFDIKRNAA.
UniRef100_A7MQ13        ..NGDTVGVTYAFQAAKVKQQYWEQTATG....GKGAETSAGWNIKENKEA
UniRef100_Q4ZV44        AEHAGLPKETITLNYGRIKLEYSQQRRTDG...GSAGIVFGGWDRVAKKP.
UniRef100_Q3BMP1        GGEDRL.TENVTLHFGKFKYGFQPQDDKGA...AQGGAKEFTYDIQGVAKA
UniRef100_A3P1G9        AQDESRPRELVRLSFSRLKQEYVVQNPQG....GSGGAITATFDIKKNAA.
UniRef100_Q8PF41        GGEDRL.TENVTLHFGKFKYGFQPQDDKGA...SQGGAKEFTYDIQGVAKA
UniRef100_Q63P47        AQDESRPRELVRLSFSRLKQEYVVQNPQG....GSGGAITATFDIKKNAA.
UniRef100_Q48C28        AEHAGLPVELITLNYGRIKFEYSQQRRADG...GSAGIVSGGWDRTANKP.
UniRef100_Q21KI0        SQDNHRPTETFTITFVDVEVKYTPYDEDGN....AEAAIAVGFDTASNVKR
UniRef100_A4LM29        AQDESRPRELVRLSFSRLKQEYVVQNPQG....GSGGAITATFDIKKNAA.
UniRef100_A3NFP4        AQDESRPRELVRLSFSRLKQEYVVQNAQG....GSGGAITATFDIKKNAA.
UniRef100_A3KB44        DGLDRI.QESLTLNFRMFEVTYTKQNQKGA...PDGE.FSAGWEIAEDKEW
UniRef100_A7CMP7        ATDEARVREKVALSFSKVKQEYMVQNAQG....GSGGAITAGYDIKGNKEA
UniRef100_A1TM68        ..GSDAVLVNYAFQAAKIKTQYWEQTNQG....GKGAESSAGWNIKENKEA
UniRef100_A8GG80        ..HTDNILVKYRFQASKVNLHYWEQSASG....TKGAETKAGWDIKQNKEI
UniRef100_Q2T924        AQDESRPRELVRLSFSRLKQEYVVQNAQG....GSGGAITATFDIKKNAA.
UniRef100_A0NQ25        GSDDRI.TESVTLNFGEYKYIYTEQKPDG....SKGAAPEFAWDIAANEKK
UniRef100_A0GAP0        ..GGDECHEDVHLSFSTMKHEYFVQNALG....GSGGAVTATLYIKNNTTN
UniRef100_Q7W1H3        ..GSEAVLVNYAFQAAKVRQQYWEQTESG....GKGAESLVAWDIKQNKEI
UniRef100_Q3BTR0        GGEDRL.TENVTLHFGKFKYSFQPQDDKGK...KQGGTKDFTFNMQEVKKS
UniRef100_Q13YG2        NGDDG.IREHVRLSFAKVRQEYVVQNAQG....GSGGAVTAGYDIKGNKEA
UniRef100_Q48Q45        AEQSGFPVEVVTLNYGRIKFEYSQQRRADG...GSAGIVSGGWDRTANKP.
UniRef100_Q2P1B8        GGEDRL.TENVTLHFGKFKYSFQPQDDKGK...KQGGTKDFTFNMQEVKKS
UniRef100_Q3IWK2        ESGHDMIAEDVAISFETITYKYVVQADDHS....AGDEHEVEYNVVAAR..
UniRef100_A1KCF7        GGEDRL.TENVTLNFAEFKYEYTKQAPKGG...KDST.MTATWNIAENDGN
UniRef100_Q48E46        DQDNSFPSELIALNYGRIQLIYSKQSRKTG...QSAGQIAGGWDAISNKI.
UniRef100_A7MRP5        ..QTDTVLVSYYFQAAKVNFHYWEQSNQG....TKGAETKAGWDIKQNKEL
UniRef100_A3PPF7        ESGHDMIAEDVAISFETITYKYVVQADDHS....AGDEHEVEYNVVAAR..
UniRef100_Q0FRC9        DGD.DNCNETVIFNYGAIKVEYYKQDKTGKLSKASGDQGETKFSQVKNKAD
UniRef100_Q0FN14        EREGQELQESIGFAFENIKIKYIVQEEDHS....AGDEHEVEFDIAMGA..
UniRef100_Q7WP83        ..GSEAVLVNYAFQAAKVRQQYWEQTESG....GKGAESLVAWDIKQNKEI
UniRef100_Q6F922        VNEEGVPTEVFGLKYAAVEWTYNQQD.ING...SAKGAVTKKWSLSNNTAS
UniRef100_Q13MH0        ..GGEVCHEEVCLSFSTMKQEYFVQNALG....GSGGAVTATLYIKNNTTN
UniRef100_A3TYB1        DGD.DICEETVVIQYGAMKVTYTPQKADG....TAGTPTNKMWSRVLNEAS
UniRef100_Q2P080        VDEDGMPQEVVQLSFGAIKWTYNQQK.PEG...GVGGKAVAQWSATKNIPN
UniRef100_Q28MX3        GD...RPVETIRLNFTRMEVKYIPHDENHA....PQSPMIASYDLTTTRAA
UniRef100_A0VH35        ..SSDTVTVNYSFQAAKVKTQYWEQTNQG....GKGAESQMGFNIKENREA
UniRef100_A0GJN3        NADNG.IREQVRLSFAKVKQEYVVQNAQG....GSGGAVTAGYDIKLNKEA
```
