# Supplementary figures and images for: Structural Basis for the Secretion of EvpC: A Key Type VI Secretion System Protein from Edwardsiella tarda
Source: PLoS One. 2010 Sep 23;5(9):e12910. doi: 10.1371/journal.pone.0012910 (PMC2944823; doi:10.1371/journal.pone.0012910)

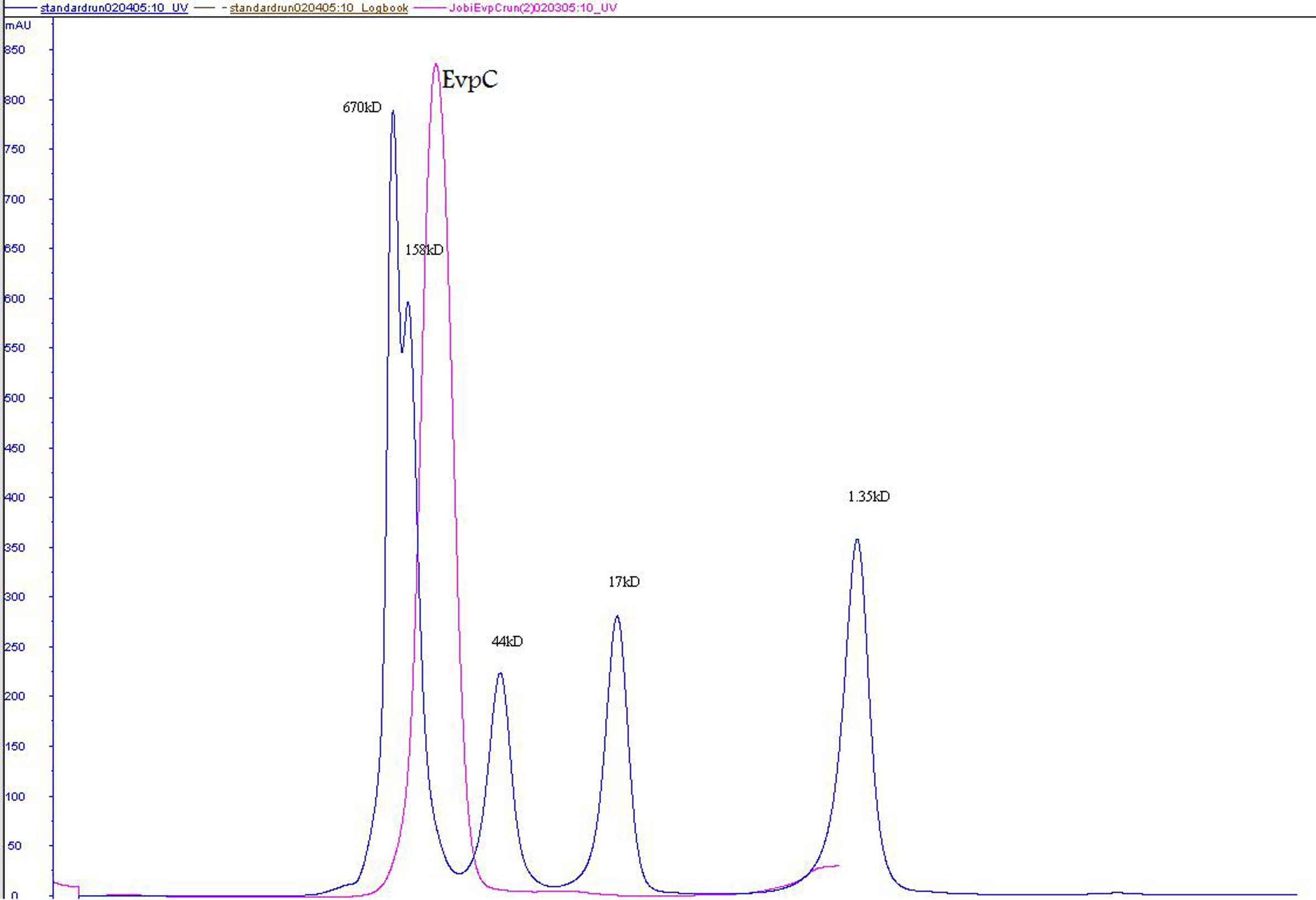

Supplement: Figure S2 — Gel filtration (Hiload16/60 Superdex 75 column) profile of EvpC at a higher concentration (>2 mg/ml), the peak corresponds to an apparent molecular weight of 120 KDa (hexamer). (1.24 MB TIF) [file pone.0012910.s003.tif]

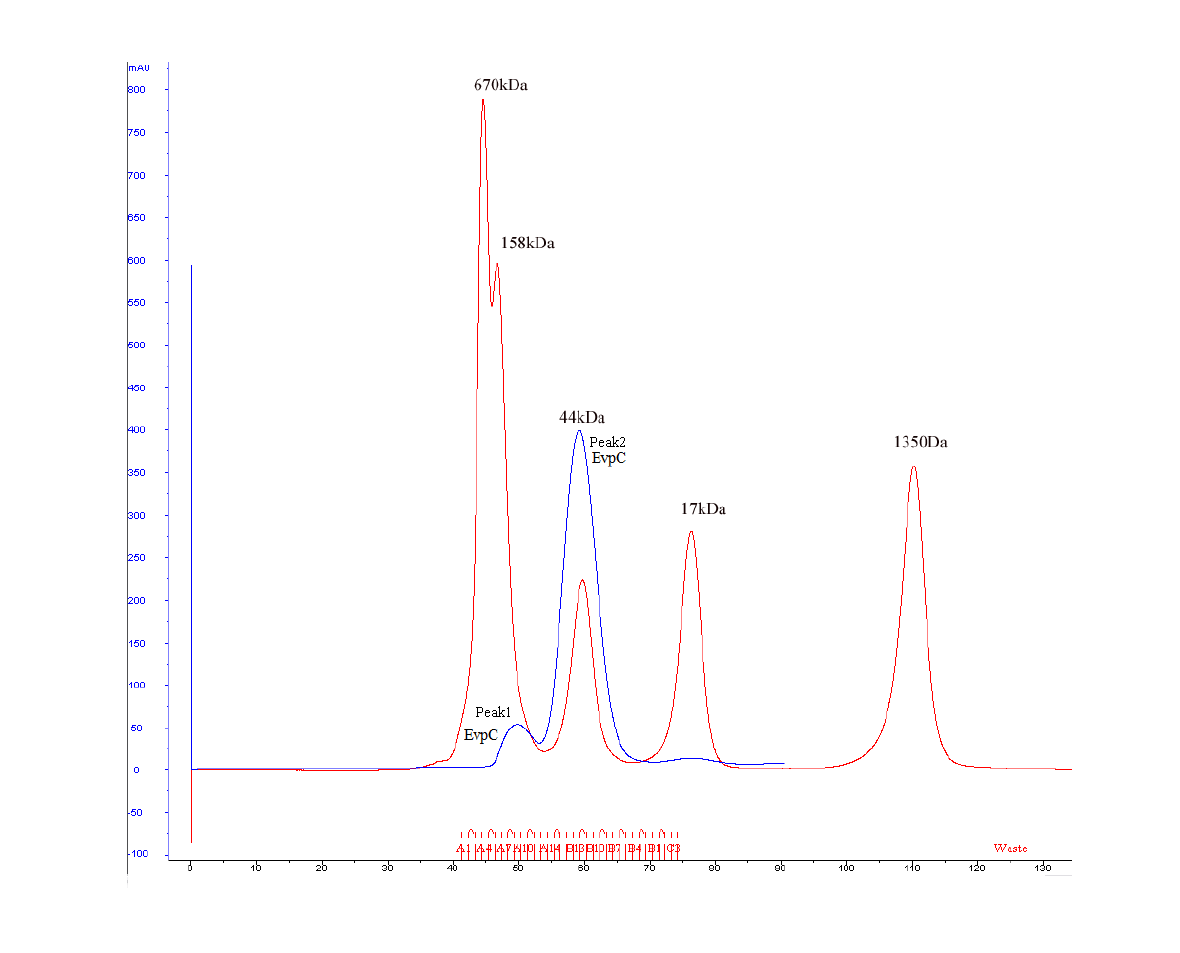

Supplement: Figure S3 — Gel filtration (Hiload16/60 Superdex 75 column) profile of EvpC at a low concentration (∼0.8 mg/ ml) shows two peaks. Peak 1 (small peak) corresponds to an apparent molecular weight of 120 kDa (hexamer) and peak 2 corresponds to an apparent molecular weight of 44 kDa (dimer). (0.11 MB TIF) [file pone.0012910.s004.tif]

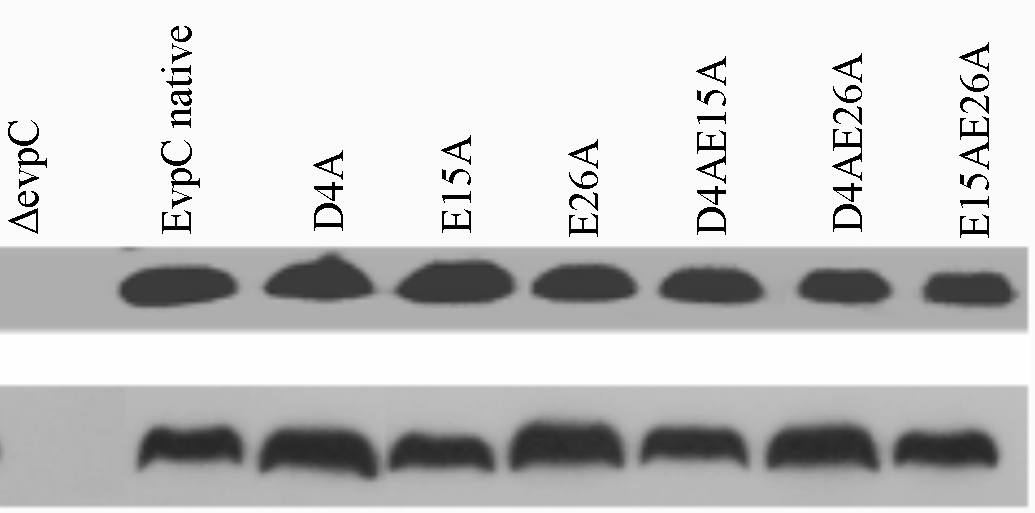

Supplement: Figure S4 — Western blots of the expression and secretion of EvpC from E. tarda. ΔevpC expressing pSA-evpC and N terminus single and double mutants of evpC. The western blot was probed with anti-EvpC rabbit polyclonal antibodies. (0.07 MB TIF) [file pone.0012910.s005.tif]

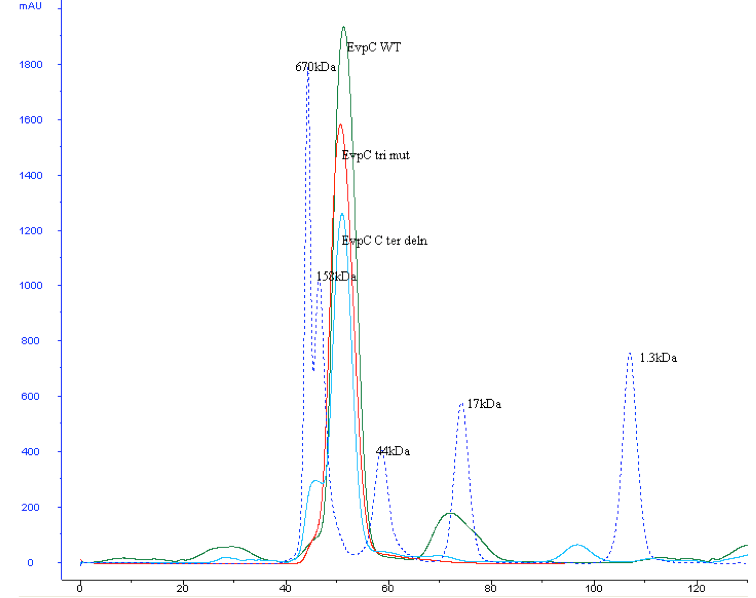

Supplement: Figure S5 — Gel filtration (Hiload16/60 Superdex 75 column) profile of EvpC wild type and mutants at 2 mg/ml concentration which shows a single peak around 120 kDa (hexameric EvpC). (0.05 MB TIF) [file pone.0012910.s006.tif]

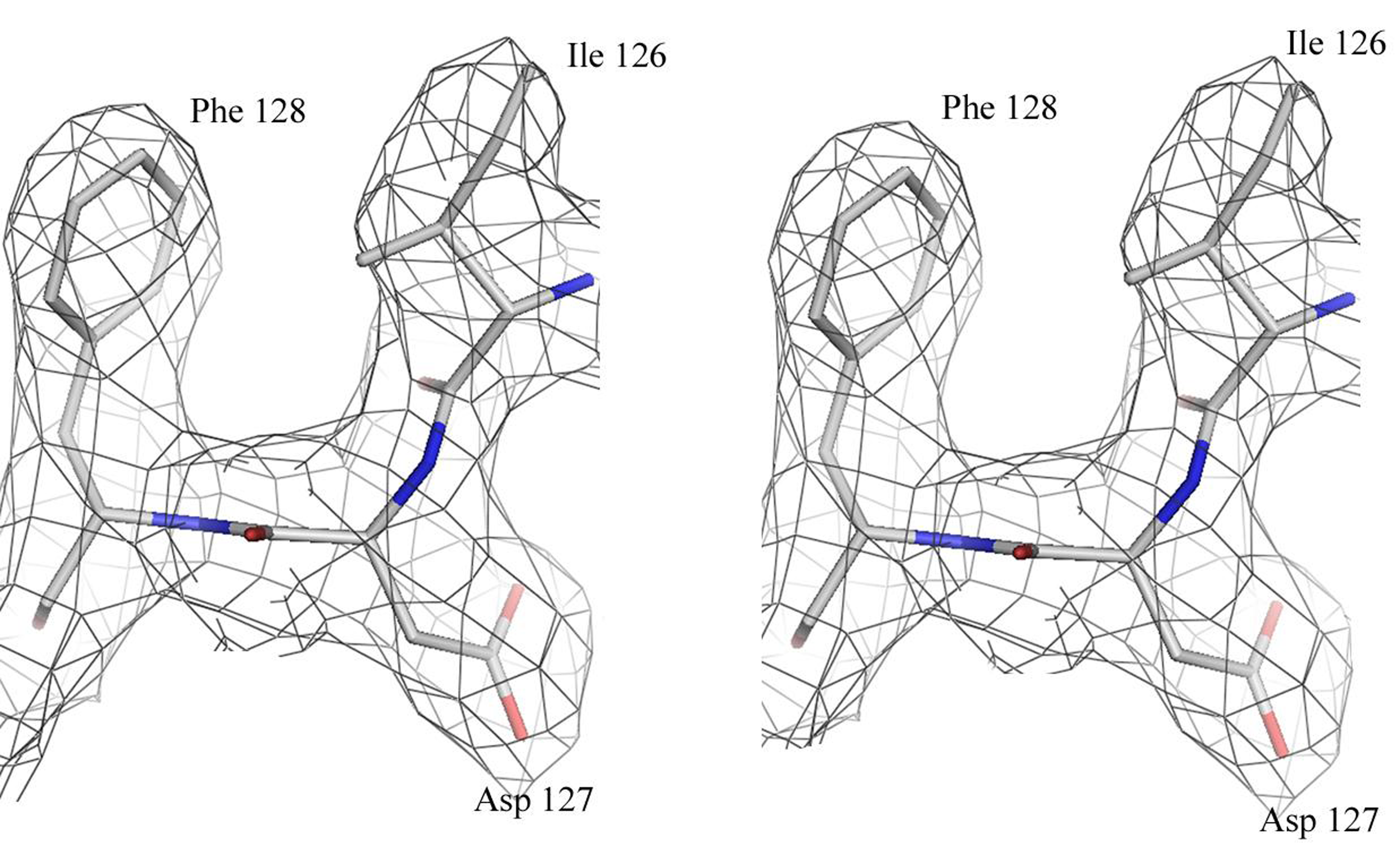

Supplement: Figure S6 — Stereo view of the final 2Fo-Fc electron density map. This map is contoured at a level of 1.0 σ. This figure was prepared using PyMol [41]. (2.55 MB TIF) [file pone.0012910.s007.tif]
